# Supplementary material for: Anesthetic-induced neurodevelopmental changes with region-specific responses to propofol in forebrain organoids
Source: Stem Cell Reports. 2026 Mar 19;21(4):102859. doi: 10.1016/j.stemcr.2026.102859 (PMC13083803; doi:10.1016/j.stemcr.2026.102859)
Supplement: Document S1. Figures S1–S4 and Tables S1 and S2 [file mmc1.pdf]

Stem Cell Reports, Volume *21*

Supplemental Information

Anesthetic-induced neurodevelopmental changes with region-specific responses to propofol in forebrain organoids

Hong-Qing She, Chang-Le Fang, Qi-Jun Li, Ruo-Lan Du, Ke-Qian Liu, Qiu-Xia Xiao, Xiao-He Tian, Wen-Yuan Wang, and Liu-Lin Xiong

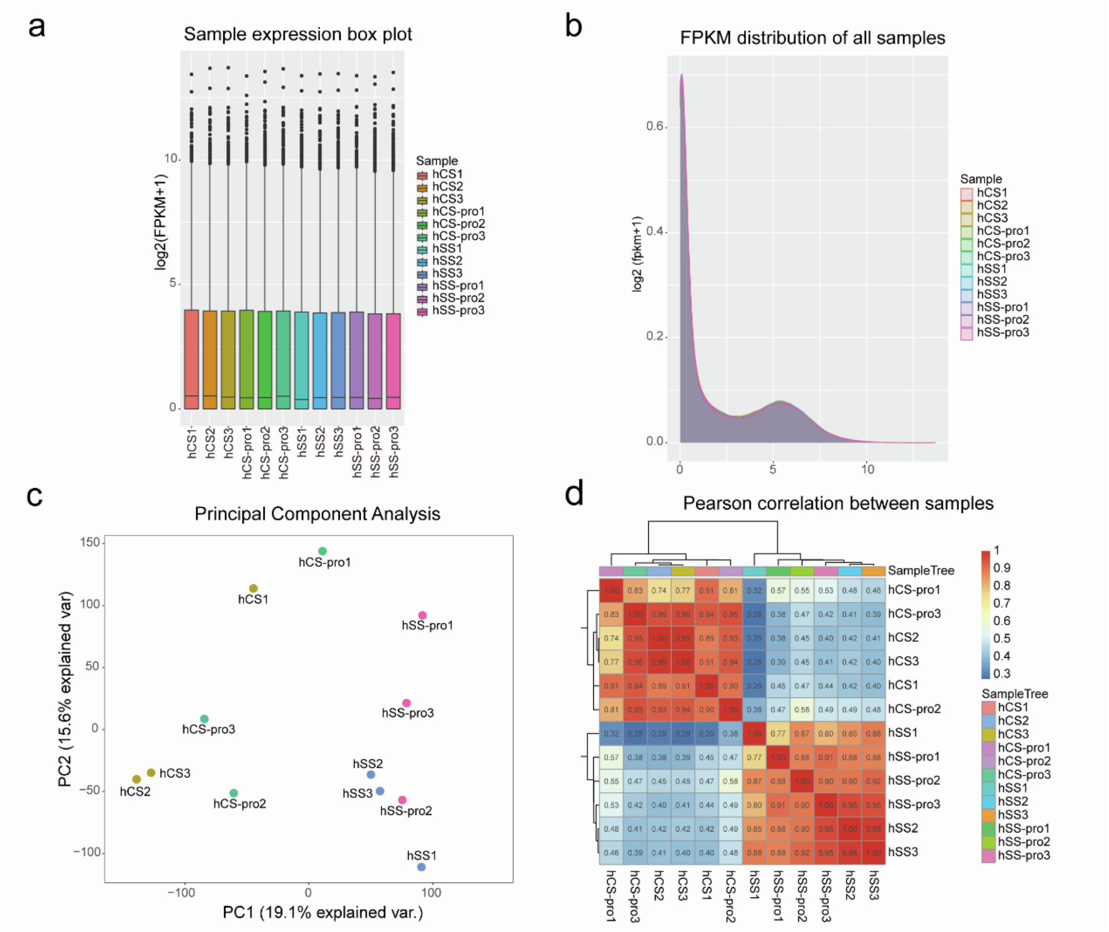

Figure S1. Gene Expression Distribution, Principal Component Analysis, and Sample Correlation Overview. (a) Box plot demonstrating the mRNA gene expression abundance in each sample. (b) Fragments Per Kilobase of transcript per Million mapped reads (FPKM) (mRNA expression abundance) distribution of all samples. (c) Principal component analysis (PCA) of the samples (based on mRNA gene expression). (d) Heatmap showing the correlation analysis of mRNA gene expression abundance in each sample.

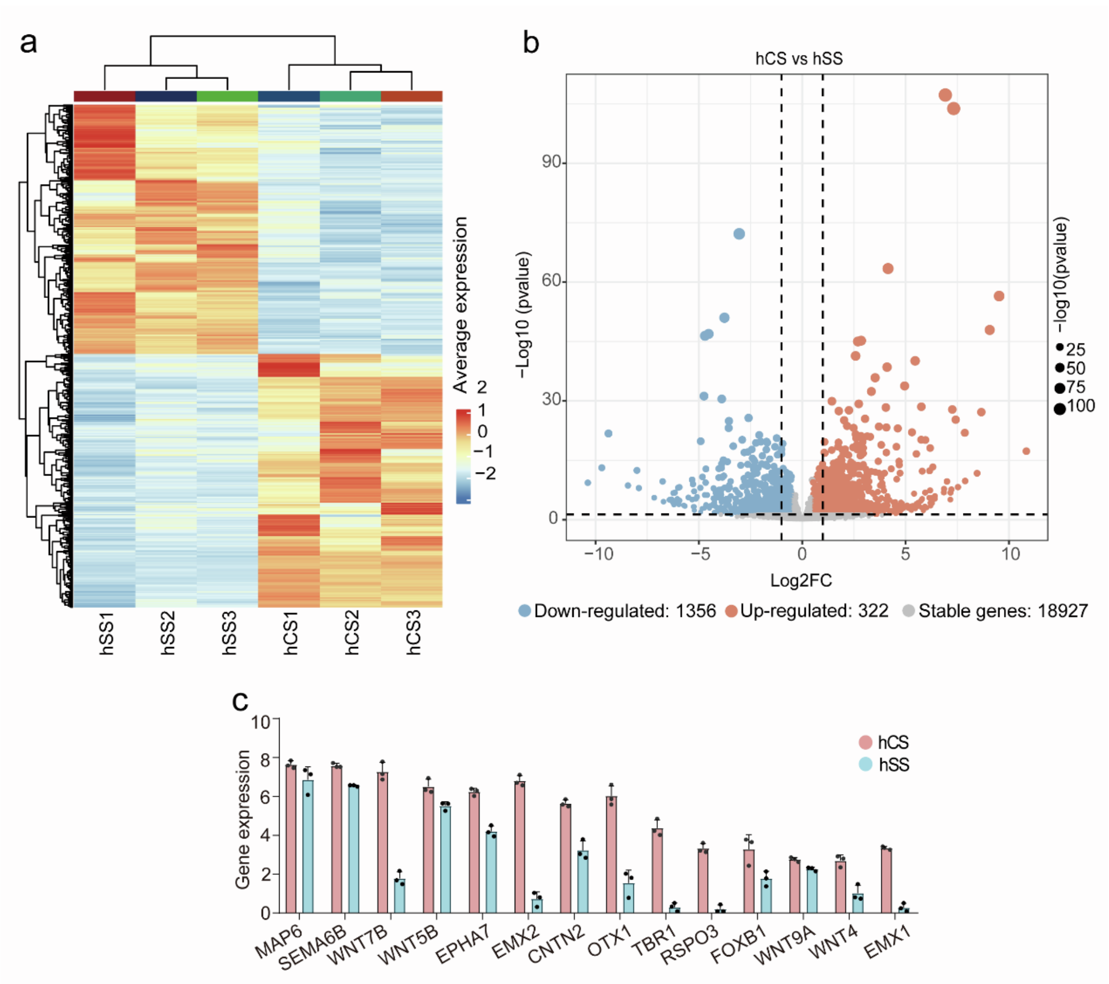

Figure S2. Differentially expressed genes between the hCS and hSS groups. (a) Heatmap showing the average expression levels of DEGs in hCS and hSS samples (red indicates high expression, blue indicates low expression). (b) Volcano plot illustrating DEGs between hCS and hSS, with upregulated genes (red), downregulated genes (blue), and stable genes (gray). (c) Bar plots displaying expression levels of dorsal forebrain markers (EMX1, EMX2, OTX1) and Wnt pathway activators (RSPO3, WNT7B, WNT5B) in hCS and hSS.

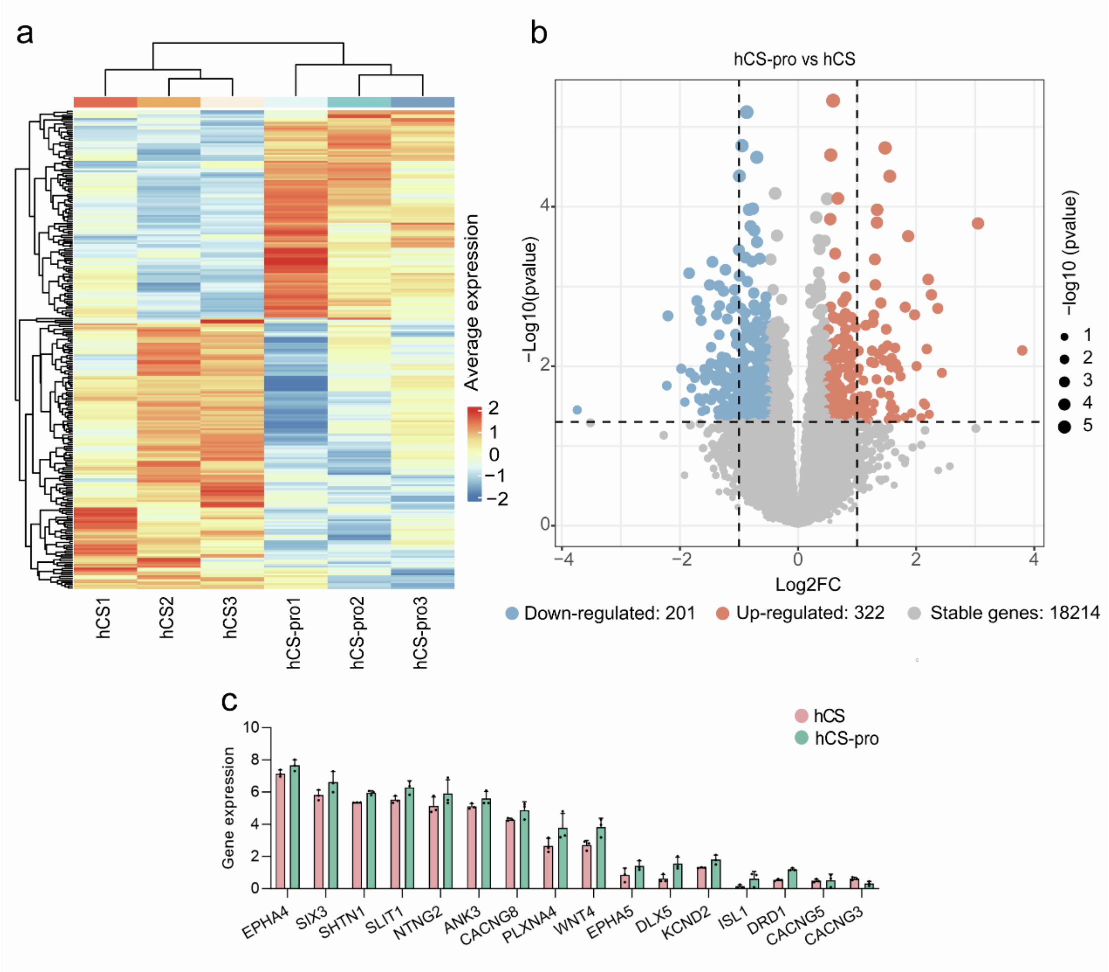

Figure S3. Differentially expressed genes between the hCS-pro and hCS groups. (a) Heatmap showing average expression levels of DEGs in hCS-pro and hCS samples (red indicates high expression, blue indicates low expression). (b) Volcano plot illustrating DEGs between hCS-pro and hCS, with upregulated genes (red), downregulated genes (blue), and stable genes (gray). (c) Bar plots displaying expression levels of key genes like DRD1, KCND2, and SLIT1 in hCS-pro and hCS.

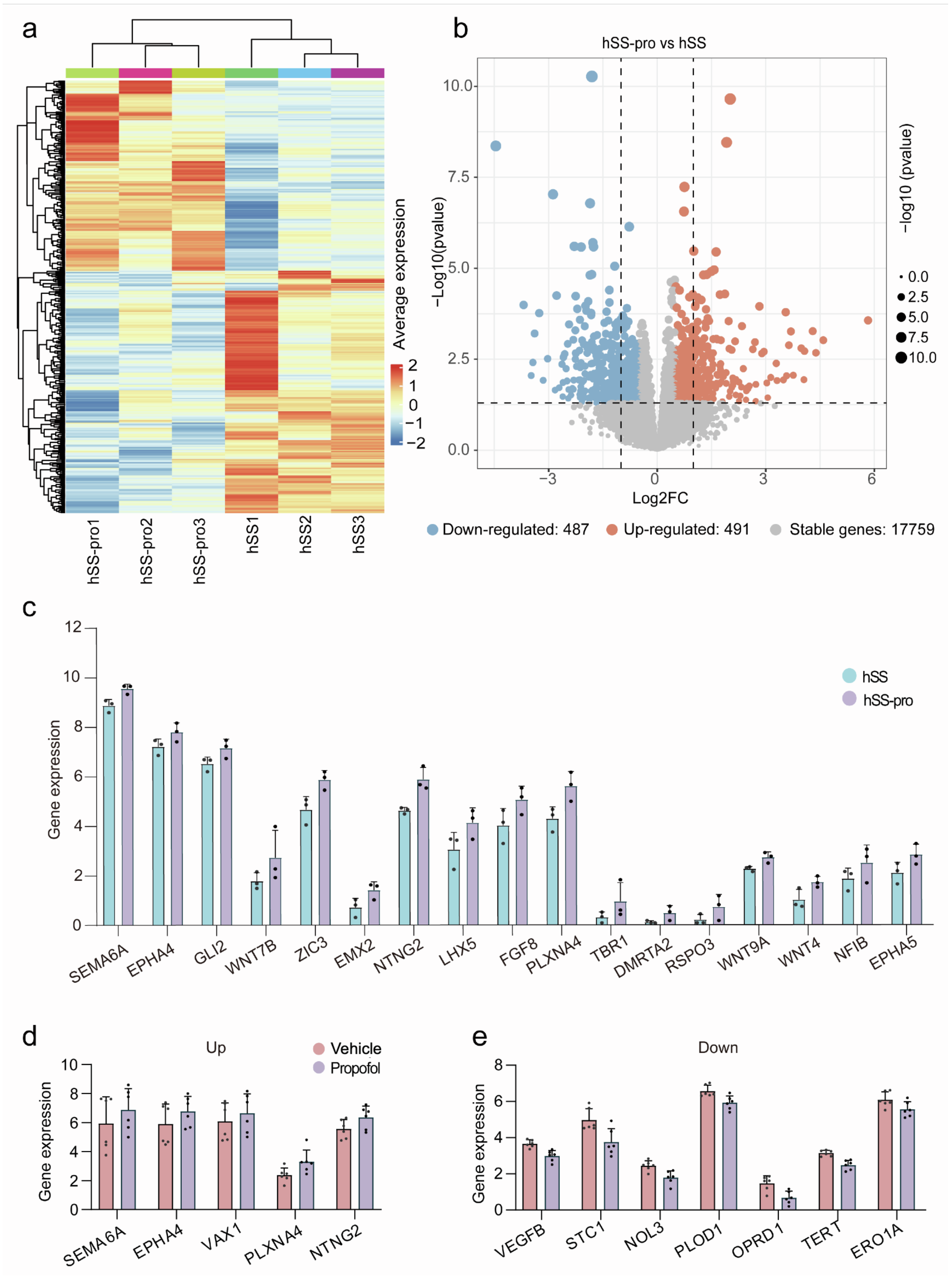

Figure S4. Differentially expressed genes between the hSS-pro and hSS groups, and between the vehicle and propofol groups.

(a) Heatmap showing average expression levels of DEGs in hSS-pro and hSS samples (red indicates high expression, blue indicates low expression). (b) Volcano plot illustrating

DEGs between hSS-pro and hSS, with upregulated genes (red), downregulated genes (blue), and stable genes (gray). (c) Bar plots displaying expression levels of key genes like FGF8, EMX2, and PAX6 in hSS-pro and hSS. (d) Expression levels of key upregulated genes, such as PLXNA4 and WNT4, involved in axon guidance. (e) Expression levels of downregulated genes, including VEGFB and PFKFB3, associated with hypoxia and metabolism.

Table

Antibody	Host	Dilution	Source	Catalog/Clone No.
Cleaved Caspase-3	Rabbit	1:400	Cell Signaling Technology	9661S
TUBB3	Mouse	1:1000	BioLegend	801201
SOX2	Mouse	1:500	R&D Systems	MAB2018
FOXG1	Rabbit	1:1000	Abcam	ab196868
NEUN	Mouse	1:200	Millipore	MAB377
MAP2	Chicken	1:500	Abcam	ab5392
NKX2.1	Rabbit	1:500	Abcam	ab76013

Supplemental Table 1

Primer	Sequence (5'→3')
SOX2-F	5'-TGGACAGTTACGCGCACAT-3'
SOX2-R	5'-CGAGTAGGACATGCTGTAGGT-3'
PAX6-F	5'-TGGGCAGGTATTACGAGACTG-3'
PAX6-R	5'-ACTCCCGCTTATACTGGGCTA-3'
MAP2-F	5'-CGAAGCGCCAATGGATTCC-3'
MAP2-R	5'-TGAACTATCCTTGCAGACACCT-3'
VGLUT1-F	5'-CAGAGTTTTCGGCTTTGCTATTG-3'
VGLUT1-R	5'-GCGACTCCGTTCTAAGGGTG-3'
GABBR2-F	5'-ACCAACTTCTTCGGGGTCAC-3'
GABBR2-R	5'-CACCTCCCTGCTGTCTTGAA-3'
VIM-F	5'-GACGCCATCAACACCGAGTT-3'
VIM-R	5'-CTTTGTCGTTGGTTAGCTGGT-3'
Caspase-3-F	5'-CCAAAGATCATACATGGAAGCG-3'
Caspase-3-R	5'-CTGAATGTTTCCCTGAGGTTTG-3'
GAPDH-F	5'-CATGAGAAGTATGACAACAGCCT-3'
GAPDH-R	5'-AGTCCTTCCACGATACCAAAGT-3'

Supplemental Table 2

Methods

Generation from hiPSC of hCS and hSS

Remove the maintenance medium from the hiPSC culture six-well plate 1 hour prior to use. Add 2 mL of Stem Flex medium (Gibco, A33493-01) supplemented with Y-27632 (10 µM; Selleck Chemicals, S1049) to each plate. Prepare the AggreWell 24-well plate by adding 1 mL of medium, supplemented with Y-27632, to each well. Centrifuge the AggreWell plate at 2,000g for 5 minutes using a swinging bucket rotor with a plate holder to remove air bubbles from the microwells.

Remove the maintenance medium from the hiPSC culture plates and wash the cells once with PBS (MesGen, MG3150-500ml). Add 1 mL of Accutase (STEMCELL Technologies, 07920) to each plate and incubate at 37°C for 6 minutes in a 5% CO_2 incubator, gently shaking the plates until the cells detach. Add 1 mL of DMEM/F12 (Gibco, 11330032) to terminate digestion and transfer the cell suspension to a centrifuge tube containing 5 mL of DMEM/F12. Centrifuge the suspension at 300g for 3 minutes. Resuspend the cell pellet in medium supplemented with Y-27632 to achieve a final concentration of 3 million cells per mL. Transfer 1 mL of this cell suspension into each well of the AggreWell plate to achieve a final volume of 2 mL per well. Centrifuge the AggreWell plate at 100g for 3 minutes. Incubate the cells at 37°C, 5% CO_2 for 26 hours.

Collect spheroids from the microwells by gently pipetting the medium up and down two to three times using a micropipette with a 1,000-µL tip. Transfer the collected spheroids to ultra-low-attachment 10-cm culture dishes. Supplement the medium with dorsomorphin (5 µM; Selleck Chemicals, s7840) and SB-431542 (10 µM; Selleck Chemicals, s1067). For

the first five days, the hiPSC medium was changed daily and supplemented with dorsomorphin and SB-431542. On the D6, neural spheroids in suspension were transferred to neural medium (NM) containing Neurobasal (Gibco, 21103049), B-27 supplement without vitamin A (Gibco, 12587001), GlutaMax (1:100; Gibco, 35050061), penicillin and streptomycin (1:100; Life Technologies, 15140122), and supplemented with growth factors EGF (20 ng/mL; NovoProtein, C029) and FGF2 (20 ng/mL; NovoProtein, C046) until D24. For the generation of hSS, the medium was supplemented with additional small molecules during the first D23 of culture. The hSS condition included the addition of the Wnt pathway inhibitor IWP-2 (5 μM; Selleckchem, S7085) from D4 to D23, and the SHH pathway agonist SAG (smoothened agonist; 100 nM; Selleckchem, S7779) from D12 to D23. AlloP (Allopregnanolone; 100 nM, Selleck, S5805) was added from D15 to D23, and there was a brief exposure (D12–15) to retinoic acid (RA, 100 nM; Sigma, R2625). The medium was changed every other day from D15 onwards. From D25 to D42, the NM for both the hCS and hSS conditions was supplemented with growth factors BDNF (20 ng/ml; NovoProtein, C076) and NT3 (20 ng/ml; NovoProtein, C079). From D43 onwards, hCS and hSS were maintained in unsupplemented NM with medium changes every four days. The hCS were cultured in the same manner as hSS, except for the addition of WP-2, SAG, AlloP, and RA.

Experimental Groups

Both hCS and hSS organoids were exposed to 20 μM propofol for 6 hours on day 11 (D11). The propofol-treated groups were compared to control groups, which were treated

with vehicle (solvent) only.

Experimental Timeline: Organoids were harvested at different time points to assess changes at different developmental stages. Morphological analyses were performed at D11, D18, and D25, while immunofluorescence and PCR were carried out at D18, D25, D40, and D80. Electrophysiological analyses were conducted between D40 and D70, and RNA-seq was performed at D18 to evaluate transcriptional changes.

Experimental Replication: The experiments were performed in biological replicates, with each condition having at least three independent organoid cultures to ensure reproducibility and reliability of the results.

Bulk RNA sequencing (RNA-Seq) and bioinformatics analysis

Four groups of brain organoids derived from hiPSCs, with a minimum of 15 organoids in each group, were analyzed. Total RNA from the brain organoids was extracted using the TRIzol reagent (Invitrogen, Thermo Fisher Scientific) and subjected to library construction and high-throughput RNA sequencing on the Illumina sequencing platform. DEGs between the groups were identified using DESeq2 (version 1.20.0). A threshold of an adjusted p-value < 0.05 and an absolute log2 fold change ≥ 0.5 was applied. Gene ontology (GO) enrichment analysis of the DEGs and statistical enrichment analysis of the Kyoto Encyclopedia of Genes and Genomes (KEGG) pathways were performed using the clusterProfiler software (version 3.8.1).

Viral labeling of neural spheroids

Briefly, hCS were transferred into a 1.5 mL microcentrifuge tube containing 400 µL of NM supplemented with 0.4 µL of recombinant adeno-associated virus (rAAV-hSyn-EGFP-WPRE-hGH pA), which expresses enhanced green fluorescent protein (EGFP) under the control of the human synapsin promoter. The spheroids were incubated overnight at 37°C with 5% CO_2 to promote viral infection. The following day, the infected neural spheroids were transferred to fresh NM.

Cryopreservation

hCS or hSS were fixed in 4% paraformaldehyde (Solarbio, P1110) for 1 hour, followed by washing with PBS. They were then transferred to a 30% sucrose solution and incubated at 4°C overnight for 48 hours. Subsequently, the spheroids were embedded in Tissue-Tek OCT compound (Sakura Finetek, 4583), snap-frozen on dry ice, and stored at -80°C. For immunohistochemistry, 20 µm thick sections were prepared using a cryostat (Leica).

Immunohistochemistry

Cryosections were washed with PBS to remove excess OCT and blocked in 5% normal goat serum (NGS, sangon biotech, E510009) and 0.3% Triton X-100 (sigma, T8787) in PBS for 1 hour at room temperature. The sections were then incubated overnight (16 hours) at 4°C with primary antibodies diluted in PBS containing 2% NGS and 0.3% Triton X-100. After washing off the primary antibodies with PBS, the cryosections were incubated with secondary antibodies diluted in PBS with 2% NGS and 0.3% Triton X-100 for 1 hour.

Following another PBS wash, the sections were stained with DAPI (1:1000 in PBS, Selleck Chemicals, S2215) for 10 minutes to visualize nuclei. The primary antibodies used were anti-Cleaved Caspase-3 (rabbit, 1:400; Cell Signaling Technology: 9661S), anti-TUBB3 (mouse, 1:1000; BioLegend: 801201), and anti-SOX2 (mouse, 1:500; R&D Systems: MAB2018). The sections were mounted on glass slides using Aquamount (Thermo Scientific) and imaged using an Olympus SpinSR10 confocal microscope. The antibodies used are listed in Supplementall Table 1.

Real time quantitative PCR (qPCR)

mRNA was isolated using Trizol, and template cDNA was synthesized by reverse transcription using the SuperScript III First-Strand Synthesis SuperMix for qRT-PCR (Thermo Fisher, 11752050). GAPDH was applied as housekeeping gene. Real-time qPCR was performed using SYBR Green (Roche) on a ViiA7 machine (Applied Biosystems, Life Technologies). Data were processed using the QuantStudio RT-PCR software (Applied Biosystems). Primers used are listed in Supplemental Table 2.

Electrophysiology

Sections of hCS (D40–70) for physiological recordings were obtained using a previously described approach. Briefly, hCS were embedded in 0.4% low-melting agarose (Takara, 5264), diluted with ACSF, and sectioned using a Leica VT1200S vibratome. Spheroids were incubated in bicarbonate-buffered artificial cerebrospinal fluid (aCSF) at 32°C and equilibrated with a mixture of 95% O_2 and 5% CO_2. The aCSF solution contained

126 mM NaCl, 26 mM $NaHCO_3$, 10 mM glucose, 2.5 mM KCl, 1.25 mM NaH_2PO_4, 1 mM $MgSO_4$, and 2 mM $CaCl_2$. Slicing was performed using a Leica VT1200S vibratome. Immediately after sectioning, slices were transferred to a circulation chamber containing oxygenated aCSF at 32°C. For patch-clamp recording, cells were identified by the presence of a fluorescent reporter using an upright BX51W1 microscope (Olympus). Recording electrodes made of borosilicate glass had a resistance of 4-9 MΩ when filled with internal solution. The low-Cl internal solution contained 145 mM K+ gluconate, 0.1 mM $CaCl_2$, 2.5 mM $MgCl_2$, 10 mM HEPES, 0.2 mM EGTA, and 4 mM Na+ phosphocreatine. Data were collected using a 1550B digitizer (Molecular Devices), and a 700B patch-clamp amplifier (Molecular Devices), and acquired with Clampex 10.7 software (Molecular Devices). Recordings were filtered at 10 kHz. APs were analyzed using Clampfit 10.7.0.3 programs.
