# Supplementary material for: Anesthetic-induced neurodevelopmental changes with region-specific responses to propofol in forebrain organoids
Source: Stem Cell Reports. 2026 Mar 19;21(4):102859. doi: 10.1016/j.stemcr.2026.102859 (PMC13083803; doi:10.1016/j.stemcr.2026.102859)
Supplement: Document S2. Article plus supplemental information [file mmc2.pdf]

STEM CELL REPORTS
Article

OPEN ACCESS

Anesthetic-induced neurodevelopmental changes with region-specific responses to propofol in forebrain organoids

Hong-Qing She,[1,3,4] Chang-Le Fang,[1,4] Qi-Jun Li,[1,4] Ruo-Lan Du,[1,2] Ke-Qian Liu,[1,2] Qiu-Xia Xiao,[1] Xiao-He Tian,[1] Wen-Yuan Wang,[1,*] and Liu-Lin Xiong[1,5,*]

[1]Department of Anesthesiology, The First People's Hospital of Zunyi (The Third Affiliated Hospital of Zunyi Medical University), Zunyi 563000, China
[2]Scientific Research Center, the Third Affiliated Hospital of Zunyi Medical University (the First People's Hospital of Zunyi), Zunyi 563000, China
[3]Interdisciplinary Research Center on Biology and Chemistry, Shanghai Institute of Organic Chemistry, Chinese Academy of Sciences, Shanghai 200032, China
[4]These authors contributed equally
[5]Lead contact
*Correspondence: wywang@sioc.ac.cn (W.-Y.W.), 499465010@qq.com (L.-L.X.)
https://doi.org/10.1016/j.stemcr.2026.102859

SUMMARY

Propofol, a common anesthetic, has unclear effects on fetal brain development. While previous studies in animal models and 2D culture systems have reported neurotoxic effects, few have addressed the region-specific vulnerability of the developing human brain. This study investigated the distinct effects of propofol on human dorsal (hCS) and ventral (hSS) forebrain organoids, uncovering region-specific differences in neuronal differentiation and maturation. Detailed electrophysiological analysis revealed that propofol enhanced neuronal activity—increased action potential frequency and amplitude—in hCS organoids. Transcriptomic analysis further indicated a metabolic shift away from hypoxic stress toward efficient aerobic pathways. These findings provide a deeper understanding of how anesthesia affects brain development, particularly in early stages, and highlight the importance of considering regional differences and long-term effects in future research.

INTRODUCTION

With growing interest in the potential effects of anesthetics on neurodevelopment, anesthesia and brain health have become critical interdisciplinary concerns in modern medicine. The fetal period represents a highly vulnerable stage of neurodevelopment, characterized by rapid neuronal proliferation, migration, and differentiation. During this time, the developing brain is particularly sensitive to external stimuli (Xu et al., 2024), including anesthetic exposure. Approximately 210 million pregnancies occur annually (Graham et al., 2016), with 1%–2% (Rasmussen et al., 2019) of these involving anesthesia-requiring nonobstetric surgery, potentially exposing the fetus to anesthetic agents. In 2016, the Food and Drug Administration issued a public warning about the potential neurodevelopmental risks associated with prolonged or repeated anesthetic exposure in children under 3 years and pregnant women, underscoring the importance of understanding the safety of these agents during pregnancy.

Propofol, one of the most widely used intravenous anesthetics, has garnered significant attention for its potential neurodevelopmental effects. Acting primarily through γ-aminobutyric acid and N-methyl-D-aspartic acid receptors (Chen et al., 2020; Yuan et al., 2021), propofol modulates the balance of excitatory and inhibitory neurotransmission. Its high lipid solubility allows it to rapidly penetrate both the blood-brain and placental barriers (Groppetti et al., 2019), raising concerns about its potential impact on fetal brain development. Preclinical studies suggest that propofol induces neurotoxic effects, including neuronal apoptosis, impaired proliferation and migration, and inhibited neurogenesis, leading to long-term deficits in cognition, memory, and learning (Cattano et al., 2008; Huang et al., 2016; Jiang et al., 2017; Liang et al., 2021; Zhang et al., 2024; Zhong et al., 2018; Zou et al., 2020). However, conflicting evidence complicates the interpretation of these findings. While some studies report significant neurotoxic effects, others indicate that short-term or low-dose exposure may not have substantial consequences for neurodevelopment (Davidson et al., 2016; McCann et al., 2019). Moreover, propofol has demonstrated neuroprotective properties under certain conditions, such as attenuating isoflurane-induced neuroinflammation (Nie et al., 2020). These divergent findings highlight the complexity of propofol's effects on fetal neurodevelopment and the need for further research to delineate its region- and time-specific impacts.

Direct studies on human fetal neurodevelopment face ethical and technical limitations, often necessitating reliance on animal models or retrospective clinical data. However, significant differences in brain structure, developmental timelines, and complexity between humans and rodents limit the translational relevance of animal studies. Recent advancements in human brain organoid technology provide a novel platform to overcome these challenges (Fang et al., 2025; Han, 2025; Luo et al., 2023; Paşca et al., 2022; Sidhaye and Knoblich, 2021; Xiong and Wang, 2024). Organoids recapitulate key aspects of

 | © 2026 The Authors.
Published by Elsevier Inc. on behalf of International Society for Stem Cell Research.
This is an open access article under the CC BY license (http://creativecommons.org/licenses/by/4.0/).

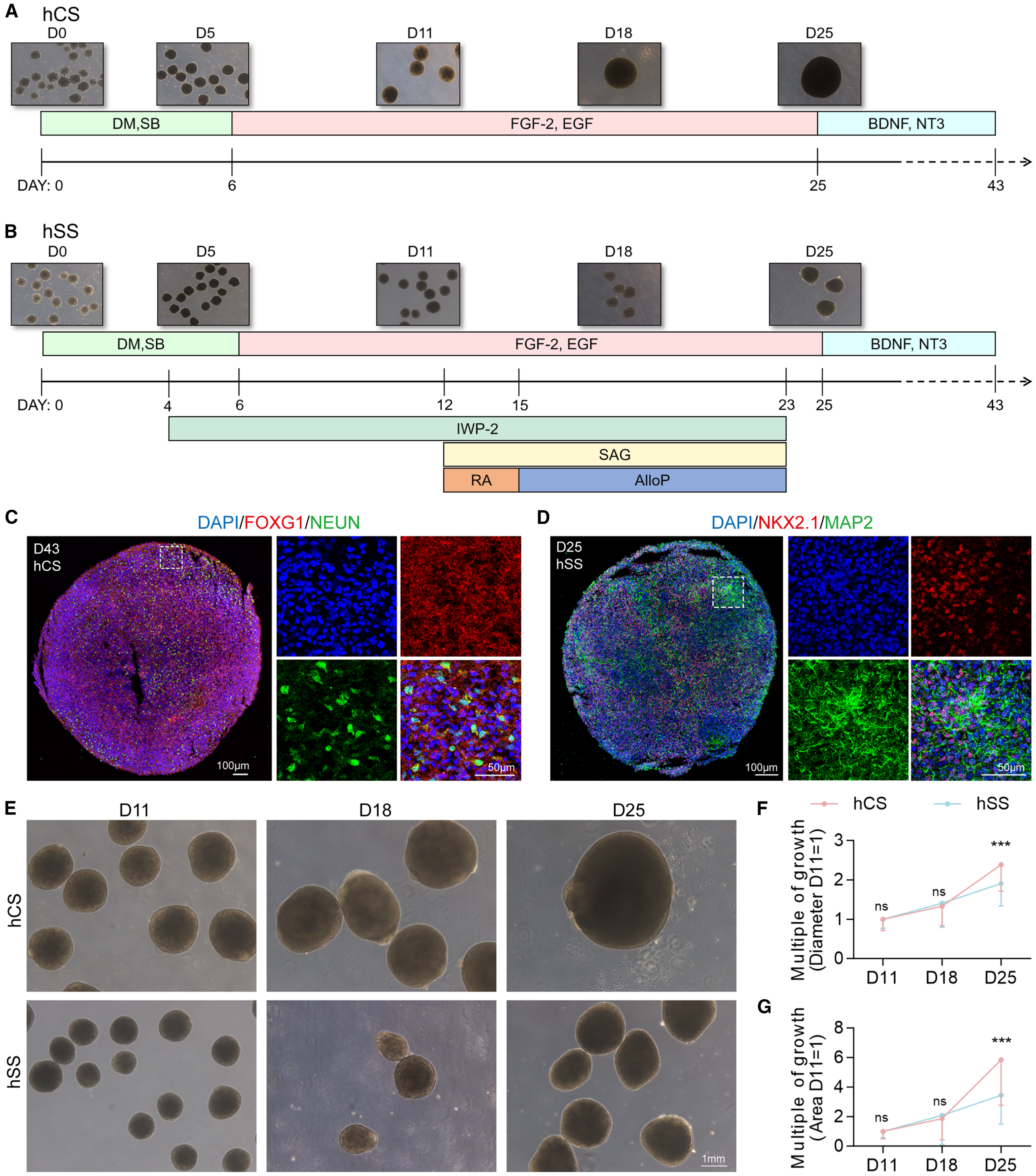

Figure 1. Development and characterization of hCS and hSS organoids from hiPSCs

(A and B) Schematic representation of the differentiation process for hCS and hSS organoids, with size progression shown across days. Organoid size increases over time (data from 29 to 99 organoids per group).

(C) Immunofluorescence staining of FOXG1 (red) and NEUN (green) in hCS at D43. Scale bars: 100 μm and 50 μm.

(D) Immunofluorescence staining of NKX2.1 (red) and MAP2 (green) in hSS at D25. Scale bars: 100 μm and 50 μm.

(legend continued on next page)

human brain development in a three-dimensional *in vitro* environment, allowing detailed investigation of cellular processes, molecular pathways, and drug effects. Among these, forebrain organoids closely mimic early-stage human forebrain development, offering an ideal model for studying neurodevelopmental toxicity (Paşca et al., 2015). By capturing the regional and temporal diversity of human brain development, forebrain organoids enable region-specific evaluations of anesthetic effects. Recent studies have begun to employ brain organoids to investigate anesthetic effects, with many focusing on anesthetic-induced neurotoxicity and its impact on neurodevelopment (Logan et al., 2020; Pant et al., 2025; Saglam-Metiner et al., 2024; Xiao et al., 2024). However, our study offers a fresh perspective by examining region-specific responses of brain organoids to propofol, along with its electrophysiological effects and the differential outcomes under varying anesthetic exposure conditions. Moreover, by providing an in-depth analysis of the genetic and phenotypic responses to anesthetic exposure, our work contributes new mechanistic insights, allowing for a comparison with existing clinical and animal studies to highlight both similarities and differences.

In this study, we employed human dorsal forebrain organoids (hCS) and human ventral forebrain organoids (hSS) to investigate the region-specific and time-dependent effects of propofol on fetal neurodevelopment. By examining its impact on excitatory and inhibitory neuronal populations, synaptic function, and metabolic adaptation, we aim to provide new insights into the clinical implications of anesthetic exposure during early pregnancy and demonstrate the utility of human brain organoids as a translational model for studying fetal brain development.

RESULTS

hCS exhibited faster growth and larger size compared to hSS

To mimic key features of early neural development, we generated three-dimensional (3D) organoid models that are self-assembling, self-organizing, and self-maturing, based on previously established protocols (Birey et al., 2017). Human induced pluripotent stem cells (hiPSCs), derived from the peripheral blood cells of two healthy donors, were differentiated into hCS and hSS. Over time, the diameter and surface area of the organoids increased progressively during culture (Figures 1A and 1B), reflecting their continuous development and maturation.

Immunofluorescence staining was performed to confirm the successful differentiation into forebrain-specific organoids. At day (D) 43 (D43), hCS expressed the dorsal forebrain marker FOXG1 and the neuronal marker NEUN, demonstrating cortical identity (Figure 1C). Meanwhile, hSS expressed the ventral forebrain marker NKX2.1 and the mature neuronal marker MAP2 (Yoon et al., 2019) as early as D25, consistent with the differentiation timeline for ventral forebrain structures (Figure 1D). These results validate the generation of region-specific forebrain organoids that recapitulate key aspects of human forebrain development.

Interestingly, hCS exhibited a faster growth rate and a larger size compared to hSS during the culture period. Bright-field microscopy images revealed clear differences in the development of hCS and hSS over time, with hCS showing a more rapid increase in diameter and surface area (Figure 1E). To quantitatively compare these differences, the diameter and surface area of the organoids were normalized to their measurements at D11. hCS showed significantly faster growth than hSS, as indicated by both diameter and surface area measurements (Figures 1F and 1G). At D25, the normalized diameter of hCS was longer (2.383 ± 0.668) compared to that of hSS (1.906 ± 0.570, $p < 0.001$) and the normalized surface area of hCS (5.861 ± 2.975) was larger than that of hSS (3.645 ± 2.098, $p < 0.001$).

These observations highlight distinct developmental trajectories for the two types of forebrain organoids. The higher growth rate and larger volume of hCS may reflect inherent differences in the developmental programs of dorsal and ventral forebrain regions. Such differences underscore the biological complexity and region-specific characteristics of neural development in the human forebrain.

Effect of propofol on organoid morphology and growth rates

To investigate the impact of propofol exposure on the growth and development of forebrain organoids, hCS and hSS were exposed to 20 μM propofol for 6 h on D11 (Figures 2A and 2B). Following this intervention, the overall morphological characteristics of organoids were assessed, and their growth rates were compared with those of unexposed control groups. Representative bright-field images of the organoids at D11, D18, and D25 revealed

(E) Representative bright-field images of hCS and hSS at D11, D18, and D25. Scale bar, 1 mm.

(F and G) Growth comparisons based on diameter and surface area, normalized to D11 values.

Data were presented as mean ± SD and analyzed by unpaired two-tailed t test (F and G) ($n > 29$ organoids per group from at least three independent differentiations). Asterisks indicate statistical significance as follows: ***$p < 0.001$. In this figure, hCS group indicates normal human dorsal forebrain organoids and hSS group indicates normal human ventral forebrain organoids. ns, no significance.

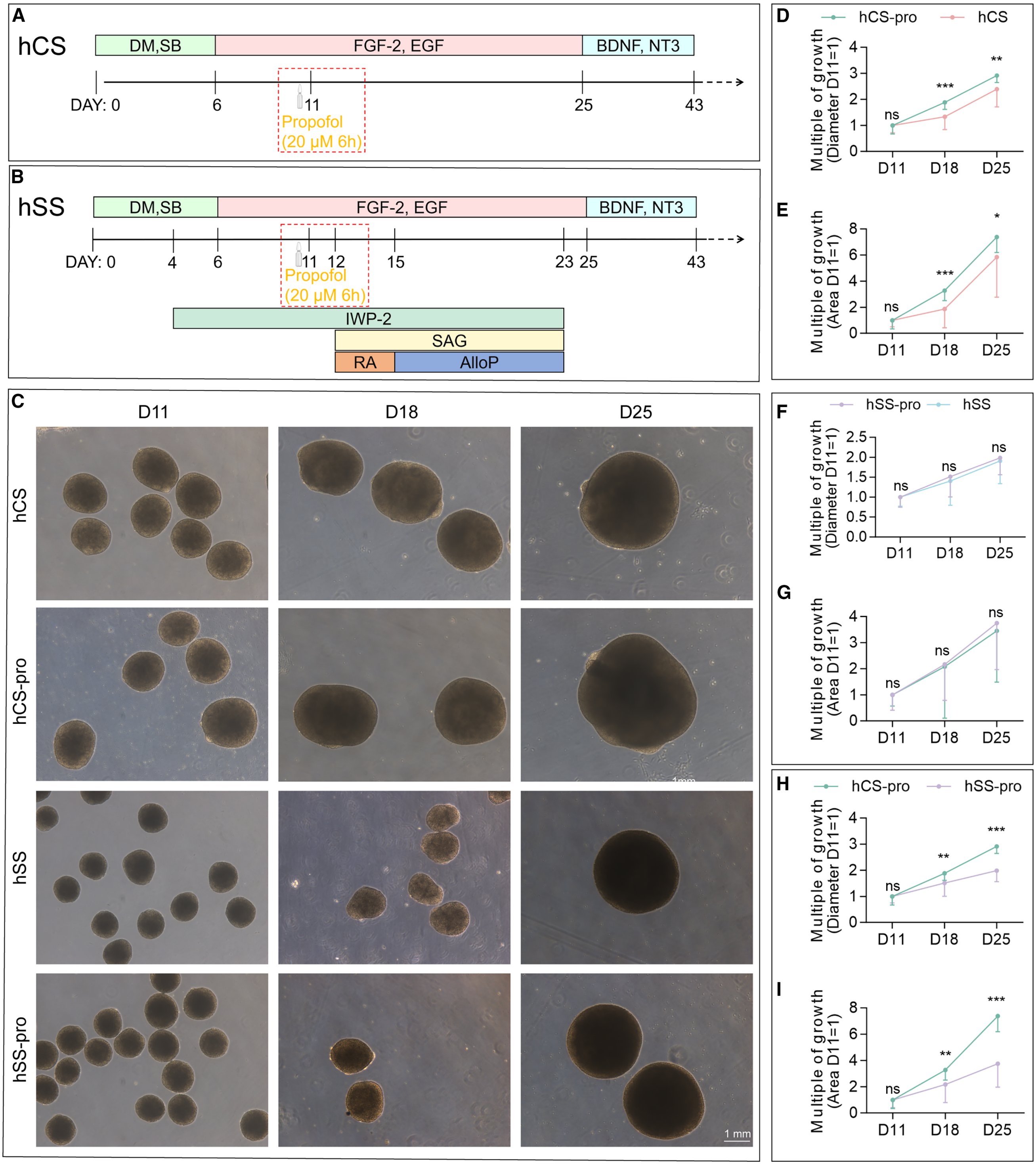

Figure 2. Effects of propofol intervention on the growth and morphology of hCS and hSS organoids

(A and B) Schematic representation of the time points for the differentiation of hCS and hSS organoids from hiPSCs, highlighting the propofol intervention on D11.

(C) Representative bright-field images of hCS, hCS-pro (propofol-exposed), hSS, and hSS-pro at D11, D18, and D25. Scale bar, 1 mm.

(D and E) Growth rates of hCS-pro and hCS in diameter (D) and surface area (E) at D18 and D25, normalized to D11 values.

(F and G) Growth rates of hSS-pro and hSS in diameter (F) and surface area (G) at D18 and D25, normalized to D11 values.

(legend continued on next page)

clear differences in size and growth trajectories between the experimental groups (Figure 2C).

To quantitatively evaluate the effect of propofol on growth, the diameter and surface area of the organoids at D18 and D25 were normalized to D11 values (baseline = 1). In hCS, propofol exposure resulted in significantly greater diameter and surface area compared to the control group at both time points (Figures 2D and 2E). At D25, propofol-exposed group (hCS-pro) exhibited a significantly larger normalized diameter (2.914 ± 0.243 vs. 2.383 ± 0.668, $p < 0.01$) and normalized surface area (7.385 ± 1.122 vs. 5.861 ± 2.975, $p < 0.05$) compared with hCS controls. This suggests that propofol exposure strongly enhanced the growth of hCS organoids. In contrast, hSS organoids showed no significant changes in diameter or surface area following propofol exposure, indicating that propofol did not affect the growth of hSS (Figures 2F and 2G). To further compare region-specific responses to propofol, growth multiples of hCS and hSS were quantified following exposure. At both D18 and D25, hCS organoids exhibited significantly greater increases in normalized diameter and surface area than hSS under propofol treatment (Figures 2H and 2I).

These results demonstrate that propofol selectively promotes the growth of hCS organoids, with no significant effect on hSS organoids. This region-specific response underscores the distinct developmental trajectories of hCS and hSS organoids and suggests that propofol's effects on neurodevelopment may be regionally specific.

Effects of propofol on organoid development, differentiation, and apoptosis of hCS and hSS at different time points

Immunofluorescence at D80 revealed reductions in cleaved caspase-3 and SOX2 in both organoid types after propofol exposure (Figure 3A), suggesting no excessive apoptosis and potential promotion of neural differentiation (Ning et al., 2019). A decrease in cleaved caspase-3 protein level, as detected by immunofluorescence, is a widely accepted indicator of reduced apoptotic activity. To strengthen this finding, we complemented it with qPCR data showing reduced caspase-3 mRNA expression (Figures 3E and 3J). Together, these consistent results at both protein and transcript levels support the conclusion of reduced apoptosis. TUBB3 expression remained unchanged, indicating unaffected mature neuron generation.

qPCR analyses further detailed the time-dependent effects of propofol on stemness, apoptosis, and differentiation markers at early (D18/25), middle (D40), and late (D80) stages. In hCS organoids (Figures 3B–3G), propofol significantly altered the expression of multiple key markers with statistical significance ($p < 0.05$) across different developmental stages. For stem cell/progenitor markers, VIM expression in hCS-pro was significantly reduced relative to the control group, with fold changes of 0.4623 at D18, 0.5020 at D40, and 0.3824 at D80 (Figure 3B). PAX6 showed consistent significant downregulation at these time points (fold changes: 0.5118 at D18, 0.5082 at D40, and 0.5049 at D80) (Figure 3C), collectively indicating a stable decline in hCS stem cells. Another stem cell marker, SOX2, exhibiting stage-specific significant reduction, was non-significant at D18 but notably downregulated at D40 (fold change: 0.2969) and D80 (fold change: 0.5014) (Figure 3D). The apoptosis-related marker caspase-3 displayed a progressive, significant downregulation across all stages (fold changes: 0.2884 at D18, 0.3671 at D40, 0.7623 at D80) (Figure 3E), suggesting that propofol lowers apoptosis rates of hCS organoids. Among neuronal function-related markers, the mature neuronal marker MAP2 maintained stable expression across all stages (Figure 3F); in contrast, the excitatory synapse marker VGLUT1 showed progressive, significant reduction relative to the control throughout development (Figure 3G). Collectively, these findings demonstrate that propofol exerts lasting effects on the expression of stem cell, apoptosis, and excitatory neuronal markers in hCS organoids.

In hSS organoids (Figures 3H–3L), propofol induced a distinct pattern with statistically significant effects ($p < 0.05$) on key marker expression. For the stem cell/progenitor marker VIM, expression remained non-significant at the D18 stage but decreased significantly at the D40 (fold change: 0.5076) and D80 (fold change: 0.4187) (Figure 3H). Stem cell marker SOX2 showed significant reductions at D18 (fold change: 0.6331), and this decreasing trend persisted into D40 (fold change: 0.2375) and D80 (fold change: 0.5674) (Figure 3I). The apoptosis marker caspase-3 exhibited stage-specific significant changes—its expression was lower at D18 (fold change: 0.3864) and D80 (fold change: 0.7334), while no significant difference was observed at the D40 (Figure 3J). The mature neuronal marker MAP2 maintained stable expression without statistical significance across all developmental stages (Figure 3K). Notably, GABBR2, an inhibitory synapse marker, showed only a transient significant reduction at the D18 (fold change: 0.6197) and returned to non-significant baseline

(H and I) Comparison of diameter (H) and surface area (I) between hCS and hSS under propofol exposure at D11, D18, and D25.
Data were presented as mean ± SD and analyzed by unpaired two-tailed t test (D–I) ($n > 29$ organoids per group from at least three independent differentiations). Asterisks indicate statistical significance as follows: *$p < 0.05$, **$p < 0.01$, ***$p < 0.001$. In this figure, hCS group indicates vehicle exposed, hSS group indicates vehicle exposed, hCS-pro group indicates propofol exposed, and hSS-pro group indicates propofol exposed. ns, no significance.

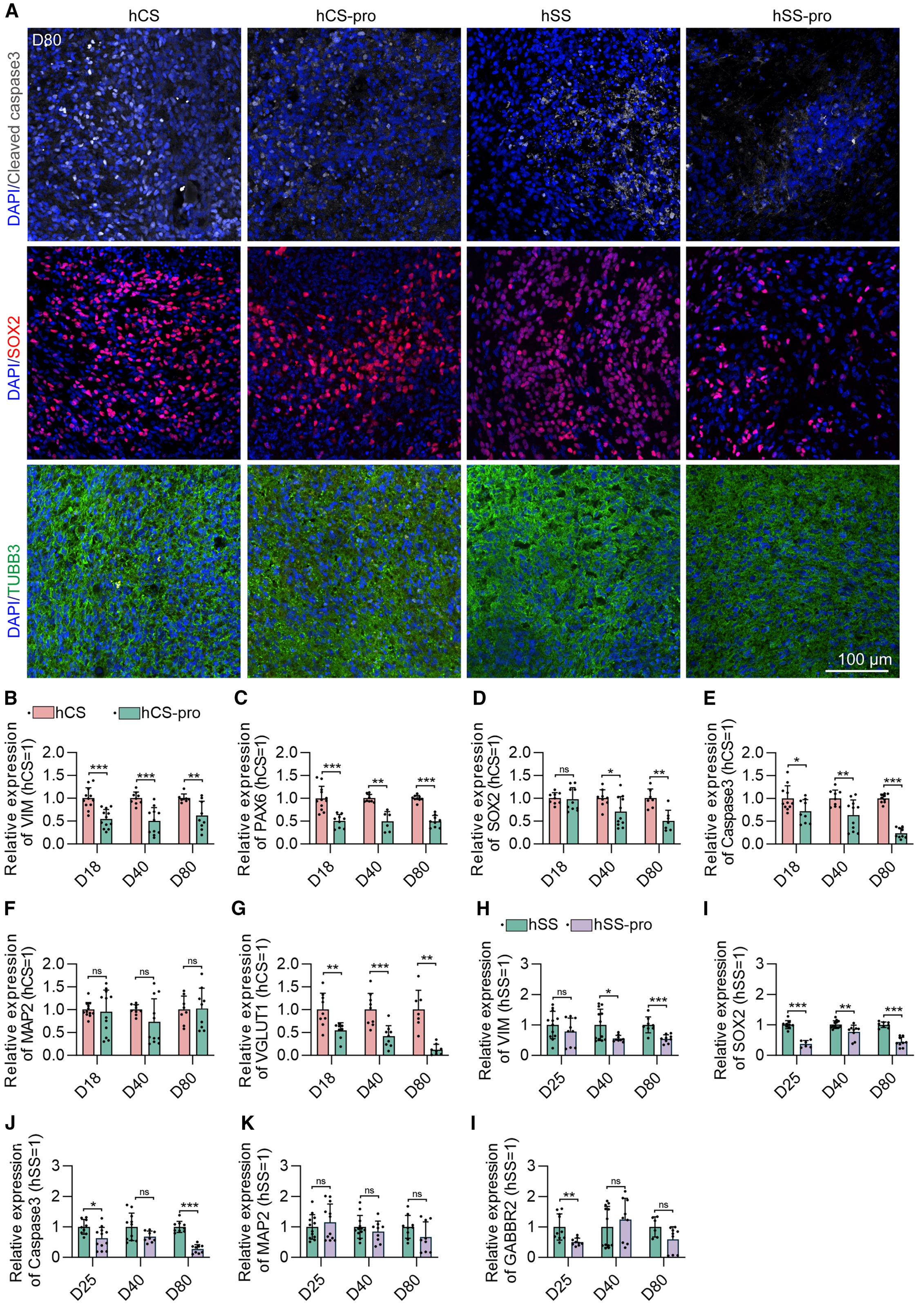

(legend on next page)

levels by the D40, with no significant change observed at the D80 (Figure 3L), indicating a recovery in inhibitory neuronal markers. Propofol exposure had consistent effects on stemness, proliferation, and apoptosis of neural progenitor cells across hCS and hSS organoids. However, region-specific differences were observed in neuronal differentiation. In hCS organoids, excitatory neuron markers were reduced at all stages, suggesting persistent effects on excitatory neuronal populations. In contrast, in hSS organoids, inhibitory neuron markers were only transiently affected during early stages, with recovery in later development. These findings underscore the region-specific responses of hCS and hSS organoids to propofol and highlight its differential effects on excitatory versus inhibitory neuronal populations.

Propofol enhances the maturation of electrophysiological function in hCS

To determine whether propofol affects the electrophysiological properties of hCS organoids, particularly in light of the observed reduction in VGLUT1 expression, we conducted patch-clamp recordings on 250 μm-thick hCS slices from D40–D70 organoids (Figure 4A). EGFP-positive neurons were identified and targeted for electrophysiological analysis (Figure 4B).

Whole-cell current-clamp recordings revealed three distinct action potential (AP) firing patterns: no AP, single AP, and multiple APs (Figure 4C). Notably, multiple APs were exclusively observed in neurons from the hCS-pro. Statistical analysis revealed a significant increase in the proportion of neurons exhibiting AP firing in the hCS-pro group compared to the control group (Figure 4D). The percentage of neurons firing APs was significantly higher in the hCS-pro group (92.3%, 24/26 cells) than in the control group (50.0%, 12/24 cells) ($\chi^2 = 11.08$, $p < 0.01$).

To further characterize neuronal excitability, neurons were stimulated with stepwise current injections ranging from −10 to 80 pA. The hCS-pro group demonstrated a significantly higher number of evoked APs compared to the control group across a range of current intensities (Figure 4E). Additionally, the amplitude of the first AP under 80-pA stimulation was significantly greater in the propofol-exposed group than in the control group (40.36 ± 12.84 vs. 25.39 ± 8.24 mV, $p < 0.01$) (Figure 4F). In contrast, no significant differences were observed in the AP half-width between the two groups (Figure 4G).

When evaluating the total number of APs generated during current stimulation, the hCS-pro group exhibited an overall increase compared to the control group, although this difference did not reach statistical significance (Figure 4H). The apparent outlier in the hCS-pro data for Figure 4G (half-width) and Figure 4H (total APs) likely represents biological variation within the heterogeneous neuronal population. All recorded cells passed standard electrophysiological quality controls (stable access resistance and seal integrity).

In summary, despite a reduction in VGLUT1 expression, propofol-exposed hCS organoids exhibited increased AP frequency and amplitude, indicating enhanced neuronal excitability and functional maturation. These findings suggest that propofol may facilitate neuronal adaptation through alternative mechanisms, promoting electrophysiological maturation in hCS organoids.

Propofol alters the transcriptional profile of forebrain organoids

To investigate the molecular mechanisms underlying the phenotypic differences induced by propofol, RNA sequencing (RNA-seq) analysis was performed at D18 on organoids exposed to a single acute propofol treatment at D11. Quality control of the samples and processed data was carried out, with results shown in Figure S1. Genes with a >0.5-fold change and a q value < 0.05 were considered differentially expressed genes (DEGs). Transcriptomic comparisons between hCS and hSS showed 1,356 genes downregulated and 322 upregulated in hCS (Figures S2A and S2B). Gene Ontology (GO) functional enrichment analysis revealed DEGs linked to forebrain development, axon development, axon guidance, and Wnt signaling. Kyoto

Figure 3. Time-dependent changes in stemness, apoptosis, and differentiation markers in hCS and hSS organoids after propofol exposure

(A) Representative immunofluorescence images showing cleaved caspase-3 (apoptosis marker), SOX2 (stemness marker), and TUBB3 (neuronal marker) in hCS, hCS-pro, hSS, and hSS-pro organoids at D80. Scale bar, 100 μm.

(B–G) Reverse-transcription qPCR (RT-qPCR) analysis of VIM, PAX6, SOX2, caspase-3, MAP2, and VGLUT1 in hCS and hCS-pro organoids across early (D18), middle (D40), and late (D80) stages.

(H–L) RT-qPCR analysis of VIM, SOX2, caspase-3, MAP2, and GABBR2 in hSS and hSS-pro organoids at early (D25), middle (D40), and late (D80) stages. Expression levels were normalized to the housekeeping gene GAPDH.

Data were presented as mean ± SD and analyzed by unpaired two-tailed *t* test (B–L) (n = 6–12 organoids per group from at least three independent differentiations). Asterisks indicate statistical significance as follows: *$p < 0.05$, **$p < 0.01$, ***$p < 0.001$. In this figure, hCS group indicates vehicle exposed, hSS group indicates vehicle exposed, hCS-pro group indicates propofol exposed, and hSS-pro group indicates propofol exposed. ns, no significance.

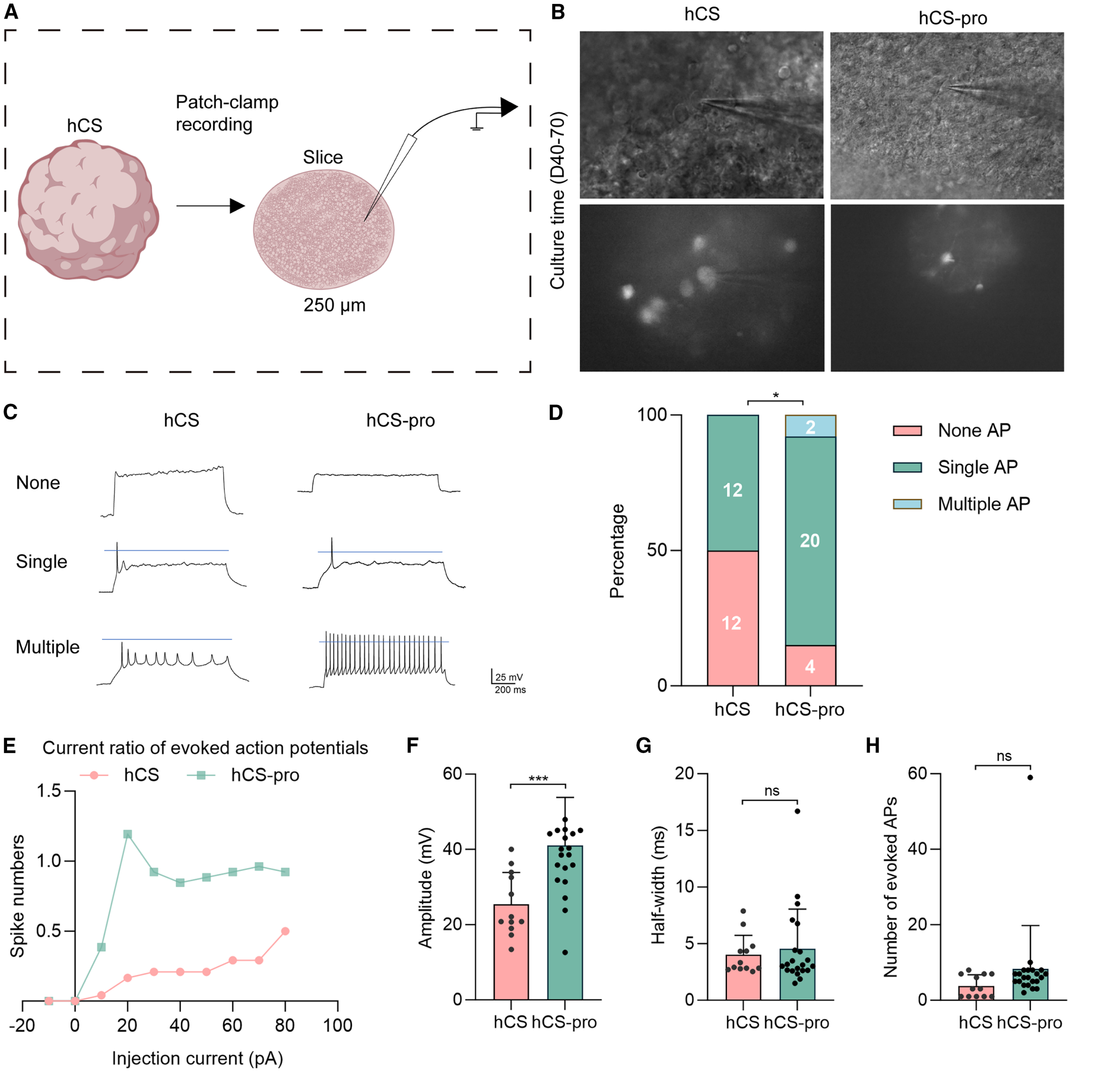

Figure 4. Electrophysiological properties of hCS and hCS-pro organoids: AP generation and amplitude

(A) Schematic representation of the hCS section for whole-cell current clamp.

(B) Representative images of EGFP-positive cells used in the electrophysiological assay.

(C) Representative traces of three whole-cell current-clamp recordings.

(D) Percentage of different AP generation patterns and the number of cells recorded in each group ($n = 24–26$ cells per group from at least three independent differentiations).

(E) Number of APs generated in hCS and hCS-pro following current stimulation (−10 to 80 pA, step 10 pA, 1 s duration).

(F–H) Quantification of the amplitude of the first AP under 80 pA current stimulation (F), AP half-width (G), and total AP number (H) ($n = 12–22$ cells per group from at least three independent differentiations). Data are presented as mean ± SD (F–H). Statistical analysis: (D) Pearson's chi-square test, $*p < 0.05$; (F) unpaired, two-tailed t test, $***p < 0.001$. In this figure, hCS group indicates vehicle exposed and hCS-pro group indicates propofol exposed. ns, no significance.

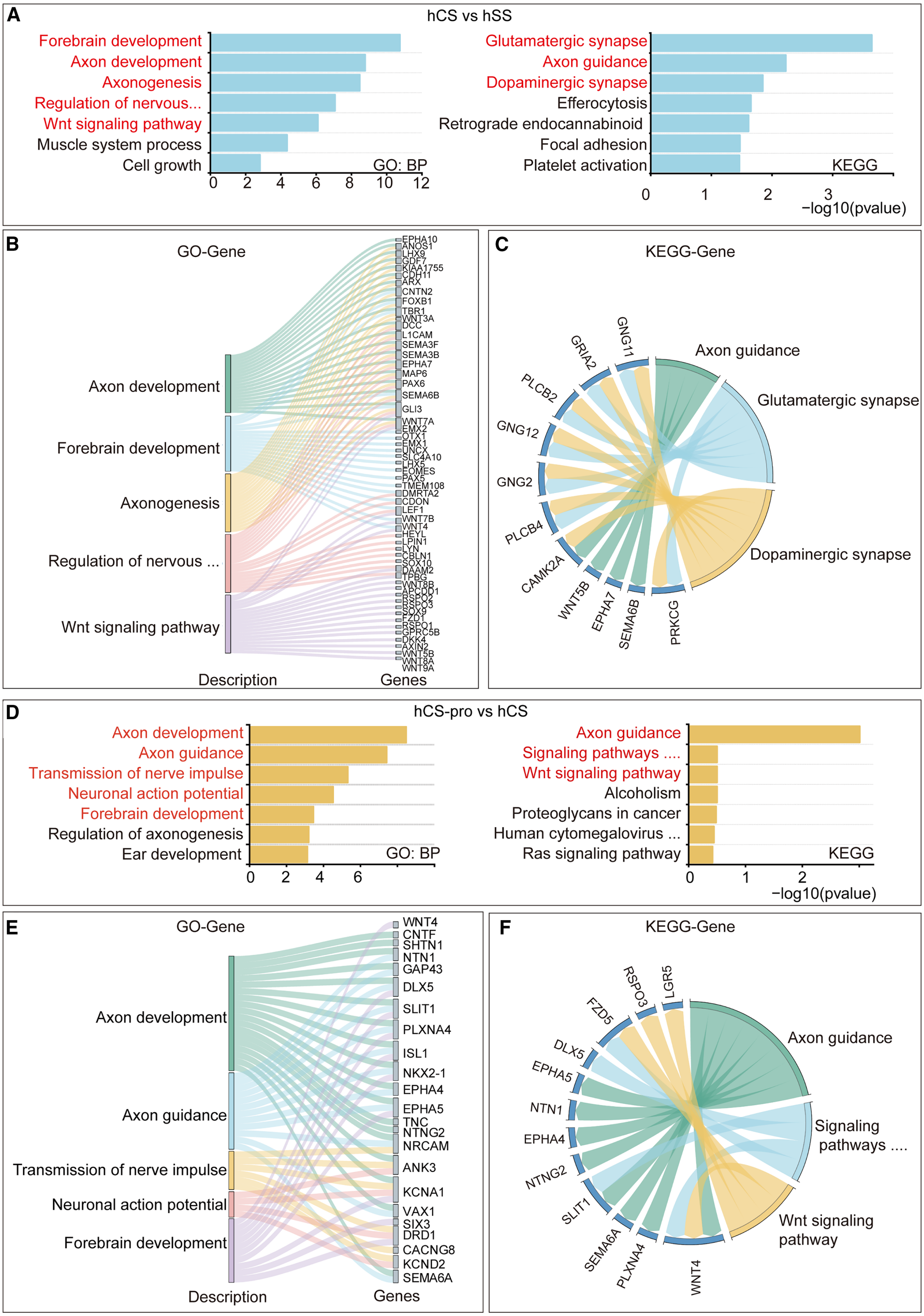

(legend on next page)

Encyclopedia of Genes and Genomes (KEGG) pathway analysis highlighted glutamatergic and dopaminergic synapse involvement (Figures 5A–5C). Key DEGs for dorsal forebrain development (RSPO3, WNT7B, and WNT5B) and cortical patterning markers (EMX1, EMX2, and OTX1) were upregulated in hCS (Figure S2C). These findings correlate with the faster growth rate of hCS organoids, indicating dorsal forebrain differentiation.

In the comparison of hCS-pro and hCS, 201 DEGs were downregulated and 322 upregulated following propofol exposure (Figures S3A and S3B). DEGs were linked to axon development, neuronal excitability, and forebrain development. KEGG analysis identified enhanced activity in axon guidance, Wnt signaling, and signaling pathways regulating pluripotency of stem cells (Figures 5D–5F). Genes like DRD1, KCND2, and SLIT1 were upregulated in hCS-pro (Figure S3C). DRD1 enhances neuronal excitability via dopamine signaling, KCND2 contributes to AP frequency, and SLIT1 aids axon guidance. These transcriptomic results align with electrophysiological findings, where hCS-pro organoids showed increased AP frequency and amplitude, suggesting enhanced neuronal maturation and connectivity. Propofol exposure upregulated pathways involved in forebrain development, axonogenesis, and AP generation, highlighting its impact on neurodevelopment and neuronal functionality.

Transcriptomic alterations and shared molecular pathways induced by propofol in forebrain organoids

Propofol exposure elicited significant transcriptomic changes in hSS organoids, with 487 genes downregulated and 491 genes upregulated compared to the control (Figures S4A and S5B). GO and KEGG pathway enrichment analyses revealed that these DEGs were involved in critical biological processes, including forebrain regionalization, central nervous system neuron differentiation, forebrain development, axon development, and axon guidance. Key signaling pathways such as Wnt signaling, Hippo signaling, and pathways regulating stem cell pluripotency were significantly enriched. Visualization through Sankey plots and KEGG chord diagrams highlighted the molecular pathways linking DEGs to developmental processes (Figures 6A–6C).

The upregulation of FGF8, a crucial regulator of forebrain regionalization, was particularly notable in hSS-pro (Figure S4C). FGF8 influences dorsal and ventral brain differentiation, increasing the expression of dorsal-specific genes such as EMX2 and PAX6, while downregulating ventral markers like NKX2.1, suggesting a shift in developmental trajectory toward dorsal characteristics. This shift aligns with the activation of the Wnt signaling pathway, typically suppressed in ventral brain development.

To identify shared molecular changes between hCS and hSS organoids, an intersection analysis was performed, revealing 52 upregulated and 50 downregulated genes common to both hCS-pro and hSS-pro groups. Upregulated genes were enriched in neuron projection guidance, axon guidance, and axon development (Figure 6D), with key genes such as PLXNA4 supporting neural network complexity and connectivity (Figure S4D). Conversely, downregulated genes were associated with cellular responses to hypoxia and metabolic pathways, including glycolysis and the HIF-1 signaling pathway (Figure 6E). Reduced expression of VEGFB, a vascular endothelial growth factor, suggests improved oxygen availability, which may mitigate hypoxic stress and enhance aerobic metabolism in organoids (Figures S4D and S4E).

Overall, propofol appears to regulate key transcriptional pathways in both hCS and hSS organoids, influencing axonal development, forebrain regionalization, and metabolic adaptation. These findings highlight its dual impact on neurodevelopmental processes and cellular environment optimization.

To validate the RNA-seq results, we performed qPCR analysis on a subset of candidate genes. Notably, EXM2 was significantly upregulated following propofol exposure ($p < 0.01$) (Figure 7), which was consistent with the transcriptomic data. This finding suggests that EXM2 may play a role in the cellular response to propofol exposure, potentially contributing to the observed neurodevelopmental alterations. Further functional studies are

Figure 5. Transcriptomic comparison between hCS-pro and hCS reveals propofol-enhanced axon development and neuronal action potentials

(A–C) Results from the comparison of hCS and hSS organoids: (A) GO biological process and KEGG pathway enrichment analyses highlight key biological processes and pathways, including forebrain development and axon guidance. (B) Sankey diagram mapping DEGs to enriched pathways such as Wnt signaling and glutamatergic synapse. (C) KEGG pathway chord plot showing relationships between key genes and pathways.

(D–F) Results from the comparison of hCS-pro and hCS organoids: (D) GO and KEGG enrichment analyses highlight significant processes and pathways, including forebrain development and axon guidance. (E) Sankey diagram mapping DEGs to pathways such as forebrain regionalization and axon development. (F) KEGG chord plot showing gene networks linked to enriched pathways. Bulk RNA-seq was performed on three independent biological replicates per group (hCS, hCS-pro, hSS, hSS-pro; $n = 3$ per group). hCS group indicates vehicle exposed, hSS group indicates vehicle exposed, and hCS-pro group indicates propofol-exposed.

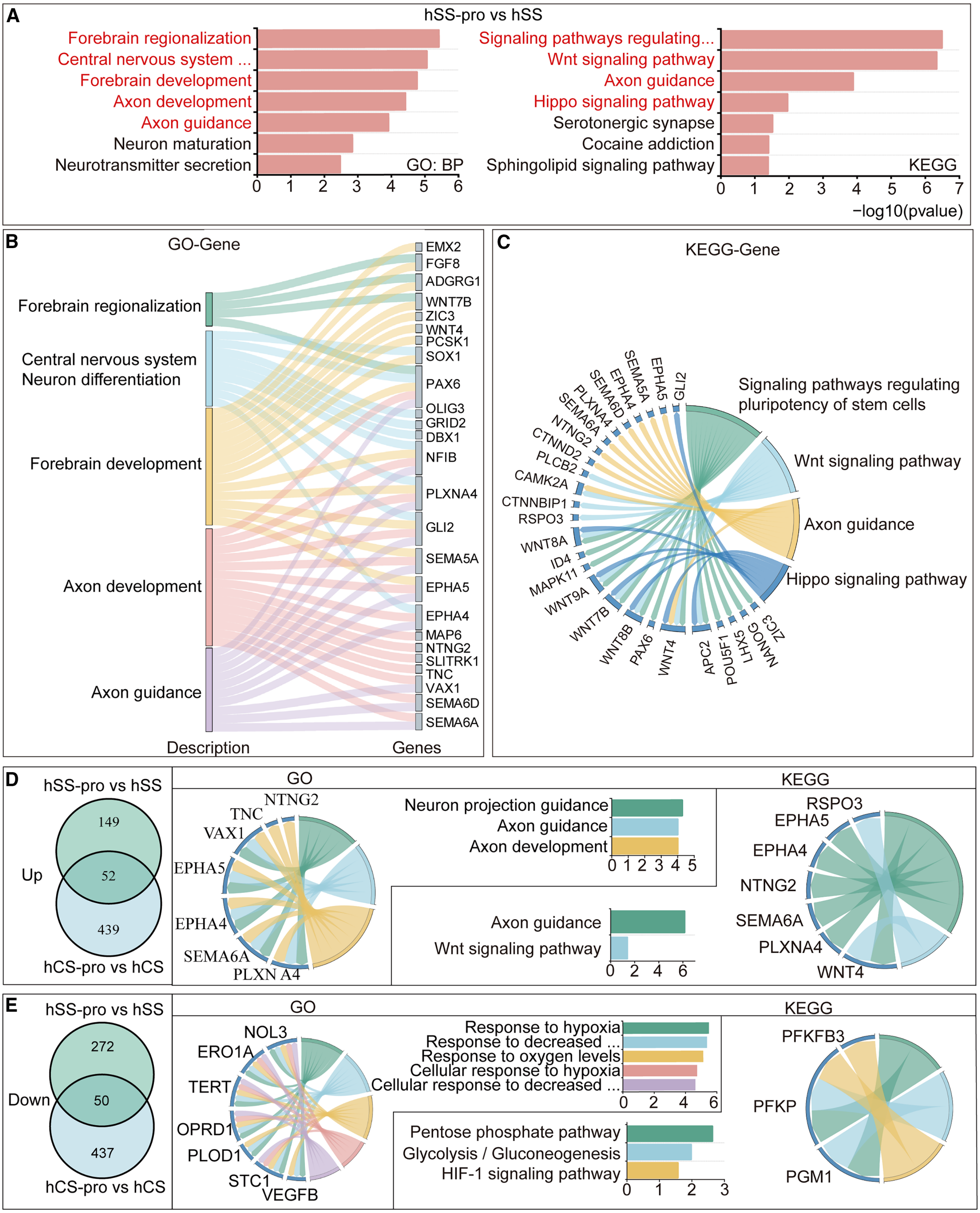

Figure 6. The common effects of propofol exposure on the transcriptomes of hSS and hCS organoids

(A–C) Results from the comparison of hSS-pro and hSS organoids: (A) GO and KEGG enrichment analyses highlight significant processes and pathways, including forebrain regionalization and axon guidance. (B) Sankey diagram mapping DEGs to pathways such as forebrain regionalization and axon development. (C) KEGG chord plot showing gene networks linked to enriched pathways. (D) Venn diagram

(legend continued on next page)

warranted to elucidate the precise mechanism by which EXM2 influences neural progenitor cell dynamics under propofol-induced conditions.

DISCUSSION

In this study, we explored the effects of propofol on human brain development, emphasizing its region-specific impact on neural differentiation and maturation. Using hCS and hSS organoids models derived from hiPSCs, we simulated the potential effects of maternal anesthesia during early pregnancy on fetal neurodevelopment. Our findings demonstrate that propofol exerts distinct, region-specific effects on brain development, uncovering key molecular pathways and mechanisms that drive these differences.

While prior studies have used brain organoids to examine general anesthetic effects, our work is distinguished by the direct comparative analysis of hCS and hSS lineages, revealing a previously unappreciated regional dichotomy in response to propofol. Our results highlight significant disparities in the developmental trajectories of hCS and hSS organoids, with hCS exhibiting a notably faster growth rate. This discrepancy reflects differences in induction protocols, including the use of the Wnt pathway inhibitor IWP-2 during hSS differentiation. Following propofol exposure, we observed accelerated growth exclusively in hCS organoids, with no comparable changes in hSS organoids. These findings suggest that the developmental impact of propofol is highly region specific and influenced by the inherent cell types and developmental stages of each brain region. The selective enhancement of hCS growth may reflect differences in the intrinsic developmental programs of dorsal and ventral forebrain regions. Propofol may interact more strongly with pathways or cellular components specific to hCS organoids, such as growth factor signaling pathways, thereby accelerating their proliferation and expansion. Alternatively, hSS organoids may be less responsive to propofol due to differences in receptor expression, cellular metabolism, or developmental timing.

In terms of neuronal differentiation, propofol induced similar effects on stemness, proliferation, and apoptosis (Hu et al., 2019; Liang et al., 2019) in both organoid types at later stages, but exhibited subtle differences at earlier stages. While the mainstream view holds that propofol increases apoptosis (He et al., 2020; Jiang et al., 2018, 2019; Logan et al., 2018), our findings indicate that it actually reduces apoptosis in organoids. We hypothesize that this discrepancy may be attributed to the absence of vasculature in the organoids, which leads to prolonged hypoxia in central cells and activates apoptosis-related pathways. Previous studies have shown that propofol alleviates hypoxia-induced decreases in cell viability and increases in apoptosis (Han et al., 2021; Hu et al., 2021; Jin et al., 2023; Jing et al., 2021; Ma et al., 2021; Xie et al., 2024), potentially by downregulating hypoxia-response genes such as VEGF (Chen et al., 2019). In our transcriptomic analysis, we further observed that propofol downregulated key metabolic pathways, including glycolysis, the pentose phosphate pathway, and the HIF-1 signaling pathway. These metabolic shifts suggest that propofol may alleviate hypoxia-induced growth restrictions by enhancing oxygen utilization and facilitating the transition to aerobic metabolism, thereby supporting neuronal development and functional integration.

Xiao et al. summarize the impact of anesthetics on fetal brain development, especially in animal and clinical studies (Xiao et al., 2024); our research adds further evidence that propofol's effects on neuronal differentiation may contradict conventional views. Moreover, a recent study using human brain organoids and pediatric serum models to investigate anesthetic-induced neurotoxicity found that prolonged exposure to propofol significantly affected neuronal development, leading to cell death and autophagy (Pant et al., 2025; Saglam-Metiner et al., 2024). Notably, the gene expression changes in the organoids were similar to those observed in pediatric serum, providing a valuable platform for translational research on anesthetic neurotoxicity. While this study emphasizes the long-term neurotoxic effects of propofol, our research complements these findings by focusing on the distinct electrophysiological and metabolic effects of propofol in organoids, highlighting the different modes of neuronal maturation under varying anesthetic exposure conditions. In a related study, a "patient-on-a-chip" platform was developed to model intensive care unit patient anesthetic responses, particularly the immune and neurotoxic effects of anesthetics (Saglam-Metiner et al., 2024). While this platform provides foundational data on anesthetic effects on the nervous system, it primarily focuses on immune responses and the effects of multiple anesthetic exposures.

showing upregulated genes common to hCS-pro vs. hCS and hSS-pro vs. hSS comparisons. GO and KEGG analyses reveal pathways related to axon guidance and Wnt signaling.
(E) Venn diagram displaying common downregulated genes. Functional analyses highlight hypoxic response and metabolic pathways. Bulk RNA-seq was performed on three independent biological replicates per group (hCS, hCS-pro, hSS, hSS-pro; $n = 3$ per group).
In this figure, hCS group indicates vehicle exposed, hSS group indicates vehicle exposed, hCS-pro group indicates propofol exposed, and hSS-pro group indicates propofol-exposed.

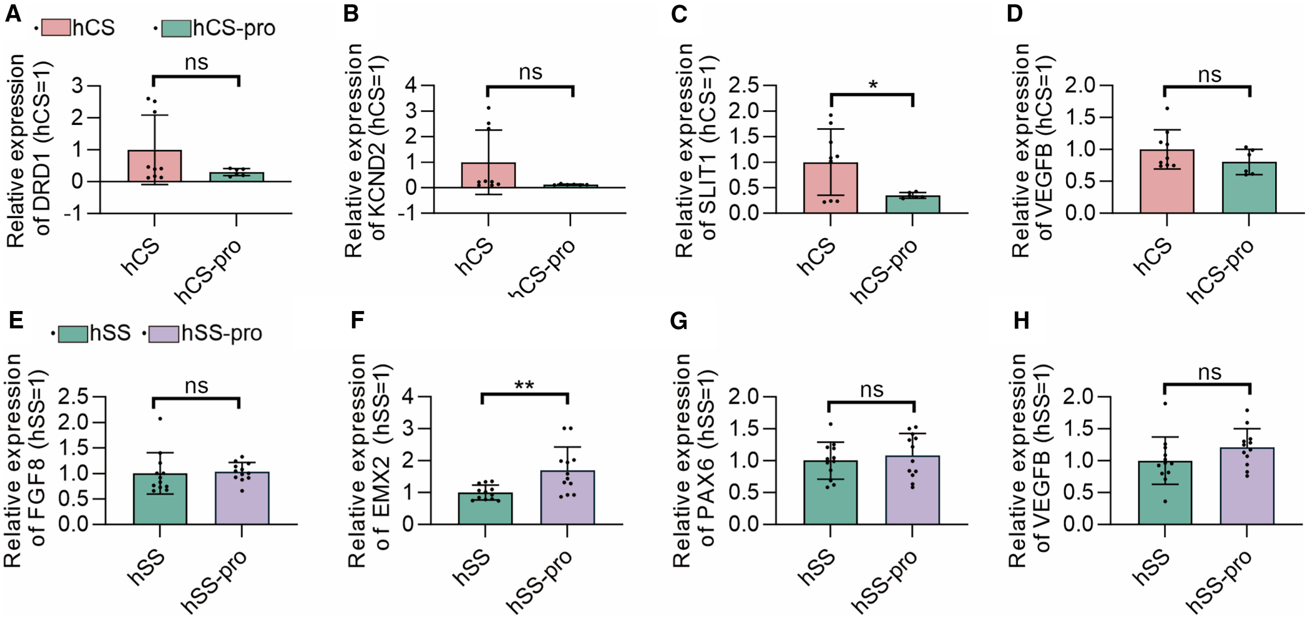

Figure 7. Propofol reduces SOX2 expression levels in hSS

(A–H) Relative mRNA expression levels of target genes in vehicle and propofol groups. qPCR was performed to assess the expression of in-vehicle and propofol-enhanced groups. Expression levels were normalized to the housekeeping gene GAPDH. Data were presented as mean ± SD and analyzed by unpaired two-tailed *t* test (A–H) (n = 6–12 organoids per group from at least three independent differentiations). Asterisks indicate statistical significance as follows: $*p < 0.05$, $**p < 0.01$. In this figure, hCS group indicates vehicle exposed, hSS group indicates vehicle exposed, hCS-pro group indicates propofol exposed, and hSS-pro group indicates propofol exposed. ns, no significance.

In contrast, our study offers an in-depth analysis of propofol's electrophysiological effects and metabolic regulation in brain organoids, demonstrating how different anesthetic exposure conditions shape neuronal maturation and functional connectivity. Together, these studies underline the importance of brain organoids as a unique model for investigating anesthetic effects on neurodevelopment. Our findings contribute new insights into the complex mechanisms by which propofol modulates neuronal differentiation and maturation, revealing the nuanced and region-specific responses of human brain organoids to anesthetic exposure. These findings have important implications for understanding the potential risks of anesthetic exposure during critical periods of neurodevelopment, particularly in vulnerable populations such as pregnant individuals.

The study of Birey et al. highlighted the electrophysiological responses of cerebral organoids to propofol exposure, marking a significant advancement in understanding how anesthetics modulate brain activity (Logan et al., 2020). In a similar vein, our work provides additional evidence that propofol exerts region- and time-specific effects on neuronal differentiation, underscoring the value of organoid models in studying anesthetic-induced neurodevelopmental changes. We observed that propofol treatment led to a consistent reduction in excitatory neuron markers, such as VGLUT1, across all stages in hCS organoids, suggesting an enduring effect on excitatory neuronal populations. In contrast, inhibitory neuron markers in hSS, such as GABBR2, were only transiently reduced at D25, with no lasting effects observed at later stages. This disruption of the excitatory-inhibitory balance raises concerns about potential neural network dysfunction, which aligns with studies linking prenatal anesthetic exposure to behavioral and cognitive impairments (Zhao et al., 2020). Electrophysiological analysis showed that propofol significantly enhanced neuronal activity in hCS organoids, with increased AP incidence and amplitude, suggesting it promotes neuronal maturation and functional connectivity. RNA-seq analysis further confirmed this, showing upregulation of genes and pathways linked to axonal development, neuronal impulse transmission, and AP generation. These electrophysiological changes not only support the idea that propofol impacts neuronal activity but also highlight its dual role in influencing both the structural and functional integration of neural circuits.

Using the machine learning algorithm CoNTExT, Pașca et al. conducted transcriptomic profiling of hCS at days 52 and 76 and compared it to adult brains at

various developmental stages. The results revealed that the transcriptomic profile of hCS closely resembles that of fetal cortical tissue, particularly in the late fetal period (19–24 post-conception weeks) (Paşca et al., 2015). This suggests that hCS can effectively model early human brain development. As organoids evolve into larger and more complex structures, and as assemblies combining region-specific organoids (e.g., thalamic and cortical organoids) are developed, the complexity of organoid connections increases (Andersen et al., 2020).

These advancements have raised ethical concerns regarding the potential for consciousness in organoids. However, current evidence strongly suggests that organoids lack the capacity for consciousness or cognition. Their morphology and function are sufficient to rule out this possibility (Farahany et al., 2018; Kataoka et al., 2023; Kosik, 2024; Lavazza and Massimini, 2018; Lunshof, 2021). Recently, human cortical organoids transplanted into rat brains have matured and integrated into neural circuits, receiving thalamocortical input and generating sensory responses. Optogenetic activation of these organoids can even drive reward-seeking behaviors, suggesting that human neurons can partially replicate sensory processing in the rat brain (Revah et al., 2022).

Consciousness remains an unresolved mystery, with no consensus on its necessary criteria (Koch et al., 2016). The rapid development of artificial intelligence (AI), neural organoids, and xenobots has amplified the need for rigorous consciousness testing. However, current methods, such as behavioral reactivity and neuroimaging, are not yet applicable to organoids due to their limited complexity (Naccache, 2018). At present, assessing consciousness in organoids remains a significant scientific and ethical challenge.

This study has several limitations inherent to the organoid model. First, although the propofol concentration used here (20 μM, approximately 3.6 μg/mL) was selected to approximate a clinically relevant unbound plasma concentration and to facilitate mechanistic investigation *in vitro*, it does not define a clinical safety threshold. The *in vivo* fetal environment during pregnancy is substantially more complex, being influenced by exposure duration, pharmacokinetics, placental transfer, and inter-individual variability. Therefore, caution is required when extrapolating these findings to clinical settings. In addition, the lack of *in vivo* features including vascularization and a functional immune system may influence cellular responses to propofol and contribute to differences from animal or clinical observations. Furthermore, the exposure paradigm was designed to model early pregnancy and may not encompass the full range of clinical scenarios. Future research should investigate propofol's effects across a spectrum of dosages and exposure durations and incorporate additional components of the neurodevelopmental milieu, such as the blood-brain barrier and glial cell interactions, to better define the translational relevance of the observed region-specific effects.

Conclusions

Propofol exerts region-specific, time-dependent effects on neural development, influencing both neuronal differentiation and metabolism. While its effects on stem cell, proliferation, and apoptosis were similar in both hCS and hSS organoids at later stages, key differences were observed in neuronal differentiation. Propofol reduced excitatory neuron markers in hCS organoids at all stages, whereas inhibitory neuron markers in hSS organoids were only transiently reduced. Electrophysiological analysis revealed that propofol enhances neuronal excitability, promoting functional maturation with increased AP generation and amplitude. Transcriptomic analysis also showed that propofol shifts cellular metabolism by reducing hypoxia-related responses and promoting aerobic pathways, likely improving oxygen supply and alleviating growth constraints. These findings highlight propofol's dual role in neuronal maturation and metabolic reprogramming. Given the potential clinical implications for pregnant women undergoing anesthesia, further studies are needed to assess the long-term neurodevelopmental consequences of propofol exposure and its impact on fetal brain development.

METHODS

Generation of hCS and hSS from hiPSCs

hCS and hSS organoids were generated and cultured using established protocols with specific medium formulations and small-molecule treatments to induce regionalization as previously described (Birey et al., 2017). Detailed procedures are provided in the supplemental information.

Bulk RNA-seq and bioinformatics analysis

RNA-seq and subsequent bioinformatics analyses, including DEGs identification, GO enrichment, and KEGG pathway analysis, were performed on brain organoids. Detailed methods are provided in the supplemental information.

Viral labeling of neural spheroids

hCS was transduced with rAAV-hSyn-EGFP to express EGFP under the human synapsin promoter. Detailed procedures are provided in the supplemental information.

Cryopreservation

hCS and hSS were fixed, cryopreserved, and sectioned for immunohistochemistry. Detailed methods are provided in the supplemental information.

Immunohistochemistry

Cryosections were stained with primary and secondary antibodies, followed by DAPI nuclear staining. Imaging was performed using confocal microscopy. The supplemental information provides detailed methods.

Real-time qPCR

Messenger RNA (mRNA) was isolated, cDNA synthesized, and real-time qPCR was performed using SYBR Green with GAPDH as a housekeeping gene. Detailed methods and primers are listed in the supplemental information.

Electrophysiology

hCS sections for physiological recordings were prepared using a vibratome and incubated in oxygenated artificial cerebrospinal fluid (ACSF). Patch-clamp recordings were conducted with specific internal solutions and analyzed using Clampfit software. Detailed methods are provided in the supplemental information.

Experimental design

This study examined the region- and time-specific effects of propofol on hCS and hSS forebrain organoids. Organoids were exposed to 20 μM propofol for 6 h on D11 and collected for RNA-seq analysis at D18. A single acute exposure was chosen to model a surgical anesthetic event during early pregnancy. The concentration of 20 μM approximates the clinically relevant unbound (free) plasma concentration of propofol during anesthesia induction and maintenance (Kawata et al., 2024; Van Hese et al., 2020). Controls were treated with vehicle. Analyses were performed at D11, D18, D25, D40, and D80, including morphological, immunofluorescence, qPCR, electrophysiological, and RNA-seq assessments. Each condition had at least three biological replicates. Details are provided in the supplemental information.

Statistical analysis

Data are presented as mean ± standard deviation (SD). All continuous variables were assessed for normality using the Shapiro-Wilk test. For comparisons between two groups, an unpaired, two-tailed Student's t test was used. Categorical data (e.g., neuron AP firing patterns) were analyzed using Pearson's chi-square test. For correlation analyses involving normally distributed numerical variables, Pearson's correlation analysis was applied. All statistical tests were two sided, with the significance level set at $\alpha = 0.05$. A p value less than 0.05 was considered statistically significant (*$p < 0.05$, **$p < 0.01$, ***$p < 0.001$). All statistical analyses were conducted with SPSS software (v.29.0; IBM Corp). Experiments were repeated with at least three independent biological replicates. Sample sizes were estimated based on previous studies and are indicated in the respective figure legends.

RESOURCE AVAILABILITY

Lead contact

Requests for further information and resources should be directed to and will be fulfilled by the lead contact, Liu-Lin Xiong (499465010@qq.com).

Materials availability

This study did not generate new unique reagents.

Data and code availability

Raw data and processed data of bulk RNA-seq are available at the GEO database with accession number GSE287509.

ACKNOWLEDGMENTS

We thank the staff members of the Integrated Laser Microscopy System at the National Facility for Protein Science in Shanghai (NFPS), Shanghai Advanced Research Institute, Chinese Academy of Sciences, China, for data collection. This work was supported by the National Natural Science Foundation of China (grant number 82560317), the Guizhou Provincial Higher Education Science and Technological Innovation Team (grant number [2023] 072), the Guizhou Province Distinguished Young Scientific and Technological Talent Program (grant number YQK [2023] 040), the Guizhou Association for Science and Technology-Young Science and Technology-Talent Lifting Project (to L.-L.X.), the Zunyi Medical University 12345 Future Talent Training Program Technology Elite (grant number: ZYSE-2021-03), and the Talent Research Startup Fund from the First People's Hospital of Zunyi (to L.-L.X.).

AUTHOR CONTRIBUTIONS

Conception and study design, H.-Q.S., C.-L.F., W.-Y.W., and L.-L.X.; acquisition of data, H.-Q.S., C.-L.F., Q.-J.L., R.-L.D., and K.-Q.L.; analysis and interpretation of data, H.-Q.S., C.-L.F., and Q.-J.L.; writing – initial draft: H.-Q.S., C.-L.F., X.-H.T., W.-Y.W., and L.-L.X.; critical revision of the manuscript for important intellectual content, H.-Q.S., C.-L.F., X.-H.T., W.-Y.W., and L.-L.X.; all authors approved of the final manuscript version to be published and agreed to be accountable for all aspects of the work, ensuring that questions related to the accuracy or integrity of any part of the work are appropriately investigated and resolved.

DECLARATION OF INTERESTS

The authors declare no competing interests.

SUPPLEMENTAL INFORMATION

Supplemental information can be found online at https://doi.org/10.1016/j.stemcr.2026.102859.

Received: September 25, 2025
Revised: February 20, 2026
Accepted: February 20, 2026
Published: March 19, 2026

REFERENCES

Andersen, J., Revah, O., Miura, Y., Thom, N., Amin, N.D., Kelley, K.W., Singh, M., Chen, X., Thete, M.V., Walczak, E.M., et al. (2020). Generation of Functional Human 3D Cortico-Motor Assembloids. Cell *183*, 1913–1929.e26. https://doi.org/10.1016/j.cell.2020.11.017.

Birey, F., Andersen, J., Makinson, C.D., Islam, S., Wei, W., Huber, N., Fan, H.C., Metzler, K.R.C., Panagiotakos, G., Thom, N., et al. (2017). Assembly of functionally integrated human forebrain spheroids. Nature *545*, 54–59. https://doi.org/10.1038/nature22330.

Cattano, D., Young, C., Straiko, M.M.W., and Olney, J.W. (2008). Subanesthetic Doses of Propofol Induce Neuroapoptosis in the Infant Mouse Brain. Anesth. Analg. *106*, 1712–1714. https://doi.org/10.1213/ane.0b013e318172ba0a.

Chen, B.P., Huang, X.X., Dong, D.M., Wu, H., Zhu, T.Q., and Wang, B.F. (2020). The role of NMDA receptors in rat propofol self-administration. BMC Anesthesiol. *20*, 149. https://doi.org/10.1186/s12871-020-01056-0.

Chen, W., Ju, X.Z., Lu, Y., Ding, X.W., Miao, C.H., and Chen, J.W. (2019). Propofol improved hypoxia-impaired integrity of blood-brain barrier via modulating the expression and phosphorylation of zonula occludens-1. CNS Neurosci. Ther. *25*, 704–713. https://doi.org/10.1111/cns.13101.

Davidson, A.J., Disma, N., de Graaff, J.C., Withington, D.E., Dorris, L., Bell, G., Stargatt, R., Bellinger, D.C., Schuster, T., Arnup, S.J., et al. (2016). Neurodevelopmental outcome at 2 years of age after general anaesthesia and awake-regional anaesthesia in infancy (GAS): an international multicentre, randomised controlled trial. Lancet *387*, 239–250. https://doi.org/10.1016/S0140-6736(15)00608-X.

Fang, C., Wang, Q., Xiao, Q., Cai, X., Du, R., Xue, L., Tian, X., and Xiong, L. (2025). Advances and applications of brain organoids in central nervous system disorders: Bridging the gap from laboratory to clinic. Neural Regen. Res. https://doi.org/10.4103/nrr.Nrr-d-24-01490.

Farahany, N.A., Greely, H.T., Hyman, S., Koch, C., Grady, C., Pașca, S.P., Sestan, N., Arlotta, P., Bernat, J.L., Ting, J., et al. (2018). The ethics of experimenting with human brain tissue. Nature *556*, 429–432. https://doi.org/10.1038/d41586-018-04813-x.

Graham, W., Woodd, S., Byass, P., Filippi, V., Gon, G., Virgo, S., Chou, D., Hounton, S., Lozano, R., Pattinson, R., and Singh, S. (2016). Diversity and divergence: the dynamic burden of poor maternal health. Lancet *388*, 2164–2175. https://doi.org/10.1016/s0140-6736(16)31533-1.

Groppetti, D., Di Cesare, F., Pecile, A., Cagnardi, P., Merlanti, R., D'Urso, E.S., Gioeni, D., Boracchi, P., and Ravasio, G. (2019). Maternal and neonatal wellbeing during elective C-section induced with a combination of propofol and dexmedetomidine: How effective is the placental barrier in dogs? Theriogenology *129*, 90–98. https://doi.org/10.1016/j.theriogenology.2019.02.019.

Han, R.H., Huang, H.M., Han, H., Chen, H., Zeng, F., Xie, X., Liu, D.Y., Cai, Y., Zhang, L.Q., Liu, X., et al. (2021). Propofol postconditioning ameliorates hypoxia/reoxygenation induced H9c2 cell apoptosis and autophagy via upregulating forkhead transcription factors under hyperglycemia. Mil. Med. Res. *8*, 58. https://doi.org/10.1186/s40779-021-00353-0.

Han, W.J. (2025). The establishment of organoids and their applications. New Discovery *4*, 1–10. https://doi.org/10.61958/ndnu9408.

He, L., Wang, X., and Zheng, S. (2020). Inhibition of the electron transport chain in propofol induced neurotoxicity in zebrafish embryos. Neurotoxicol. Teratol. *78*, 106856. https://doi.org/10.1016/j.ntt.2020.106856.

Hu, Q., Huang, L., Zhao, C., Shen, Y., Zheng, X.F., Wang, Y., Zhou, C.H., and Wu, Y.Q. (2019). Ca(2+)-PKCα-ERK1/2 signaling pathway is involved in the suppressive effect of propofol on proliferation of neural stem cells from the neonatal rat hippocampus. Brain Res. Bull. *149*, 148–155. https://doi.org/10.1016/j.brainresbull.2019.04.005.

Hu, Y., Ye, C., Cheng, S., and Chen, J. (2021). Propofol Downregulates lncRNA MALAT1 to Alleviate Cerebral Ischemia-Reperfusion Injury. Inflammation *44*, 2580–2591. https://doi.org/10.1007/s10753-021-01525-9.

Huang, J., Jing, S., Chen, X., Bao, X., Du, Z., Li, H., Yang, T., and Fan, X. (2016). Propofol Administration During Early Postnatal Life Suppresses Hippocampal Neurogenesis. Mol. Neurobiol. *53*, 1031–1044. https://doi.org/10.1007/s12035-014-9052-7.

Jiang, C., Logan, S., Yan, Y., Inagaki, Y., Arzua, T., Ma, P., Lu, S., Bosnjak, Z.J., and Bai, X. (2018). Signaling network between the dysregulated expression of microRNAs and mRNAs in propofol-induced developmental neurotoxicity in mice. Sci. Rep. *8*, 14172. https://doi.org/10.1038/s41598-018-32474-3.

Jiang, L., Yang, F., Zhao, Q., and Pan, L. (2019). MicroRNA-665 mediates propofol-induced cell apoptosis in human stem cell-derived neurons. Bioengineered *10*, 493–500. https://doi.org/10.1080/21655979.2019.1682105.

Jiang, Q., Wang, Y., and Shi, X. (2017). Propofol Inhibits Neurogenesis of Rat Neural Stem Cells by Upregulating MicroRNA-141-3p. Stem Cells Dev. *26*, 189–196. https://doi.org/10.1089/scd.2016.0257.

Jin, C., Yuan, S., Piao, L., Ren, M., and Liu, Q. (2023). Propofol synergizes with circAPBB2 to protect against hypoxia/reoxygenation-induced oxidative stress, inflammation, and apoptosis of human cardiomyocytes. Immun. Inflamm. Dis. *11*, e952. https://doi.org/10.1002/iid3.952.

Jing, H., Wang, C., Zhao, L., Cheng, J., Qin, P., and Lin, H. (2021). Propofol protects cardiomyocytes from hypoxia/reoxygenation injury via regulating MALAT1/miR-206/ATG3 axis. J. Biochem. Mol. Toxicol. *35*, e22880. https://doi.org/10.1002/jbt.22880.

Kataoka, M., Gyngell, C., Savulescu, J., and Sawai, T. (2023). The Ethics of Human Brain Organoid Transplantation in Animals. Neuroethics *16*, 27. https://doi.org/10.1007/s12152-023-09532-3.

Kawata, M., Yonezawa, A., Mineharu, Y., Itohara, K., Mizota, T., Matsui, Y., Kikuchi, T., Yamao, Y., Hattori, E.Y., Hamada, M., et al. (2024). Development of extended pharmacokinetic models for propofol based on measured blood and brain concentrations. Sci. Rep. *14*, 6326. https://doi.org/10.1038/s41598-024-56863-z.

Koch, C., Massimini, M., Boly, M., and Tononi, G. (2016). Neural correlates of consciousness: progress and problems. Nat. Rev. Neurosci. *17*, 307–321. https://doi.org/10.1038/nrn.2016.22.

Kosik, K.S. (2024). Why brain organoids are not conscious yet. Patterns (N Y) *5*, 101011. https://doi.org/10.1016/j.patter.2024.101011.

Lavazza, A., and Massimini, M. (2018). Cerebral organoids: ethical issues and consciousness assessment. J. Med. Ethics *44*, 606–610. https://doi.org/10.1136/medethics-2017-104555.

Liang, C., Du, F., Wang, J., Cang, J., and Xue, Z. (2019). Propofol Regulates Neural Stem Cell Proliferation and Differentiation via Calmodulin-Dependent Protein Kinase II/AMPK/ATF5 Signaling Axis. Anesth. Analg. *129*, 608–617. https://doi.org/10.1213/ane.0000000000003844.

Liang, C., Sun, M., Zhong, J., Miao, C., and Han, X. (2021). The Role of Pink1-Mediated Mitochondrial Pathway in Propofol-Induced Developmental Neurotoxicity. Neurochem. Res. *46*, 2226–2237. https://doi.org/10.1007/s11064-021-03359-1.

Logan, S., Arzua, T., Yan, Y., Jiang, C., Liu, X., Yu, L.K., Liu, Q.S., and Bai, X. (2020). Dynamic Characterization of Structural, Molecular, and Electrophysiological Phenotypes of Human-Induced Pluripotent Stem Cell-Derived Cerebral Organoids, and Comparison with Fetal and Adult Gene Profiles. Cells *9*, 1301. https://doi.org/10.3390/cells9051301.

Logan, S., Jiang, C., Yan, Y., Inagaki, Y., Arzua, T., and Bai, X. (2018). Propofol Alters Long Non-Coding RNA Profiles in the Neonatal Mouse Hippocampus: Implication of Novel Mechanisms in Anesthetic-Induced Developmental Neurotoxicity. Cell. Physiol. Biochem. *49*, 2496–2510. https://doi.org/10.1159/000493875.

Lunshof, J.E. (2021). Brain Surrogates-Empty or Full Makes the Difference. Am. J. Bioeth. *21*, 46–48. https://doi.org/10.1080/15265161.2020.1845872.

Luo, B.Y., Liu, K.Q., and Fan, J.S. (2023). Bibliometric analysis of cerebral organoids and diseases in the last 10 years. Ibrain *9*, 431–445. https://doi.org/10.1002/ibra.12139.

Ma, H., Liu, Y., Li, Z., Yu, L., Gao, Y., Ye, X., Yang, B., Li, H., and Shi, J. (2021). Propofol Protects Against Hepatic Ischemia Reperfusion Injury via Inhibiting Bnip3-Mediated Oxidative Stress. Inflammation *44*, 1288–1301. https://doi.org/10.1007/s10753-021-01416-z.

McCann, M.E., de Graaff, J.C., Dorris, L., Disma, N., Withington, D., Bell, G., Grobler, A., Stargatt, R., Hunt, R.W., Sheppard, S.J., et al. (2019). Neurodevelopmental outcome at 5 years of age after general anaesthesia or awake-regional anaesthesia in infancy (GAS): an international, multicentre, randomised, controlled equivalence trial. Lancet *393*, 664–677. https://doi.org/10.1016/S0140-6736(18)32485-1.

Naccache, L. (2018). Why and how access consciousness can account for phenomenal consciousness. Philos. Trans. R. Soc. Lond. B Biol. Sci. *373*, 20170357. https://doi.org/10.1098/rstb.2017.0357.

Nie, Y., Li, S., Yan, T., Ma, Y., Ni, C., Wang, H., and Zheng, H. (2020). Propofol Attenuates Isoflurane-Induced Neurotoxicity and Cognitive Impairment in Fetal and Offspring Mice. Anesth. Analg. *131*, 1616–1625. https://doi.org/10.1213/ANE.0000000000004955.

Ning, X., Wang, Y., Jing, M., Sha, M., Lv, M., Gao, P., Zhang, R., Huang, X., Feng, J.M., and Jiang, Z. (2019). Apoptotic Caspases Suppress Type I Interferon Production via the Cleavage of cGAS, MAVS, and IRF3. Mol. Cell *74*, 19–31.e7. https://doi.org/10.1016/j.molcel.2019.02.013.

Pant, T., Jiang, C., Yan, Y., Schultz, B., Laws, D., Logan, S., Bedrat, A., Juric, M., Berens, R.J., Taylor, S.P., et al. (2025). Bridging Stem Cell Models and Medicine: Integrated 3D Human Cerebral Organoids and Pediatric Serum Reveal Mechanisms and Biomarkers of Anesthetic-Induced Neurotoxicity. Preprint at bioRxiv. https://doi.org/10.1101/2025.04.23.650256.

Paşca, A.M., Sloan, S.A., Clarke, L.E., Tian, Y., Makinson, C.D., Huber, N., Kim, C.H., Park, J.Y., O'Rourke, N.A., Nguyen, K.D., et al. (2015). Functional cortical neurons and astrocytes from human pluripotent stem cells in 3D culture. Nat. Methods *12*, 671–678. https://doi.org/10.1038/nmeth.3415.

Paşca, S.P., Arlotta, P., Bateup, H.S., Camp, J.G., Cappello, S., Gage, F.H., Knoblich, J.A., Kriegstein, A.R., Lancaster, M.A., Ming, G.-L., et al. (2022). A nomenclature consensus for nervous system organoids and assembloids. Nature *609*, 907–910. https://doi.org/10.1038/s41586-022-05219-6.

Rasmussen, A.S., Christiansen, C.F., Uldbjerg, N., and Nørgaard, M. (2019). Obstetric and non-obstetric surgery during pregnancy: A 20-year Danish population-based prevalence study. BMJ Open *9*, e028136. https://doi.org/10.1136/bmjopen-2018-028136.

Revah, O., Gore, F., Kelley, K.W., Andersen, J., Sakai, N., Chen, X., Li, M.Y., Birey, F., Yang, X., Saw, N.L., et al. (2022). Maturation and circuit integration of transplanted human cortical organoids. Nature *610*, 319–326. https://doi.org/10.1038/s41586-022-05277-w.

Saglam-Metiner, P., Yanasik, S., Odabasi, Y.C., Modamio, J., Negwer, M., Biray-Avci, C., Guler, A., Erturk, A., Yildirim, E., and Yesil-Celiktas, O. (2024). ICU patient-on-a-chip emulating orchestration of mast cells and cerebral organoids in neuroinflammation. Commun. Biol. *7*, 1627. https://doi.org/10.1038/s42003-024-07313-z.

Sidhaye, J., and Knoblich, J.A. (2021). Brain organoids: an ensemble of bioassays to investigate human neurodevelopment and disease. Cell Death Differ. *28*, 52–67. https://doi.org/10.1038/s41418-020-0566-4.

Van Hese, L., Theys, T., Absalom, A.R., Rex, S., and Cuypers, E. (2020). Comparison of predicted and real propofol and remifentanil concentrations in plasma and brain tissue during target-controlled infusion: a prospective observational study. Anaesthesia *75*, 1626–1634. https://doi.org/10.1111/anae.15125.

Xiao, Q.X., Geng, M.J., Wang, Q.L., Fang, C.L., Zhang, J.H., Liu, Q., and Xiong, L.L. (2024). Unraveling the effects of prenatal anesthesia on neurodevelopment: A review of current evidence and future directions. Neurotoxicology *105*, 96–110. https://doi.org/10.1016/j.neuro.2024.09.003.

Xie, L., Zhao, M., Zong, L., and Yue, Y. (2024). Propofol Ameliorates Sepsis-Induced Myocardial Dysfunction via Anti-Apoptotic, Anti-Oxidative Properties, and mTOR Signaling. Discov. Med. *36*, 2088–2097. https://doi.org/10.24976/Discov.Med.202436189.193.

Xiong, Y.R., and Wang, T.H. (2024). Recent advances in brain organoids: a comprehensive review of the last eight years. New Discovery *2024*, 1–14. https://doi.org/10.61958/ndym9750.

Xu, L., Zhang, Y., Chen, X., Hong, Y., Zhang, X., Hu, H., Han, X., Zou, X., Xu, M., Zhu, W., and Liu, Y. (2025). Human Brain Organoids Model Abnormal Prenatal Neural Development Induced by

Thermal Stimulation. Cell Prolif. *58*, e13777. https://doi.org/10.1111/cpr.13777.

Yoon, S.J., Elahi, L.S., Pașca, A.M., Marton, R.M., Gordon, A., Revah, O., Miura, Y., Walczak, E.M., Holdgate, G.M., Fan, H.C., et al. (2019). Reliability of human cortical organoid generation. Nat. Methods *16*, 75–78. https://doi.org/10.1038/s41592-018-0255-0.

Yuan, X., Zhang, D., Mao, S., and Wang, Q. (2021). Filling the Gap in Understanding the Mechanism of GABA(A)R and Propofol Using Computational Approaches. J. Chem. Inf. Model. *61*, 1889–1901. https://doi.org/10.1021/acs.jcim.0c01290.

Zhang, W., Liu, Q., Wang, J., and Liu, L. (2024). Anaesthesia and brain development: a review of propofol-induced neurotoxicity in pediatric populations. J. Dev. Orig. Health Dis. *15*, e2. https://doi.org/10.1017/S2040174424000059.

Zhao, T., Chen, Y., Sun, Z., Shi, Z., Qin, J., Lu, J., Li, C., Ma, D., Zhou, L., and Song, X. (2020). Prenatal sevoflurane exposure causes neuronal excitatory/inhibitory imbalance in the prefrontal cortex and neurofunctional abnormality in rats. Neurobiol. Dis. *146*, 105121. https://doi.org/10.1016/j.nbd.2020.105121.

Zhong, Y., Chen, J., Li, L., Qin, Y., Wei, Y., Pan, S., Jiang, Y., Chen, J., and Xie, Y. (2018). PKA-CREB-BDNF signaling pathway mediates propofol-induced long-term learning and memory impairment in hippocampus of rats. Brain Res. *1691*, 64–74. https://doi.org/10.1016/j.brainres.2018.04.022.

Zou, L., Ning, M., Wang, W., Zheng, Y., Ma, L., and Lv, J. (2020). Naringenin Prevents Propofol Induced Neurodegeneration in Neonatal Mice Brain and Long-Term Neurocognitive Impacts on Adults. Drug Des. Devel. Ther. *14*, 5469–5482. https://doi.org/10.2147/DDDT.S280443.

Stem Cell Reports, Volume *21*

Supplemental Information

Anesthetic-induced neurodevelopmental changes with region-specific responses to propofol in forebrain organoids

Hong-Qing She, Chang-Le Fang, Qi-Jun Li, Ruo-Lan Du, Ke-Qian Liu, Qiu-Xia Xiao, Xiao-He Tian, Wen-Yuan Wang, and Liu-Lin Xiong

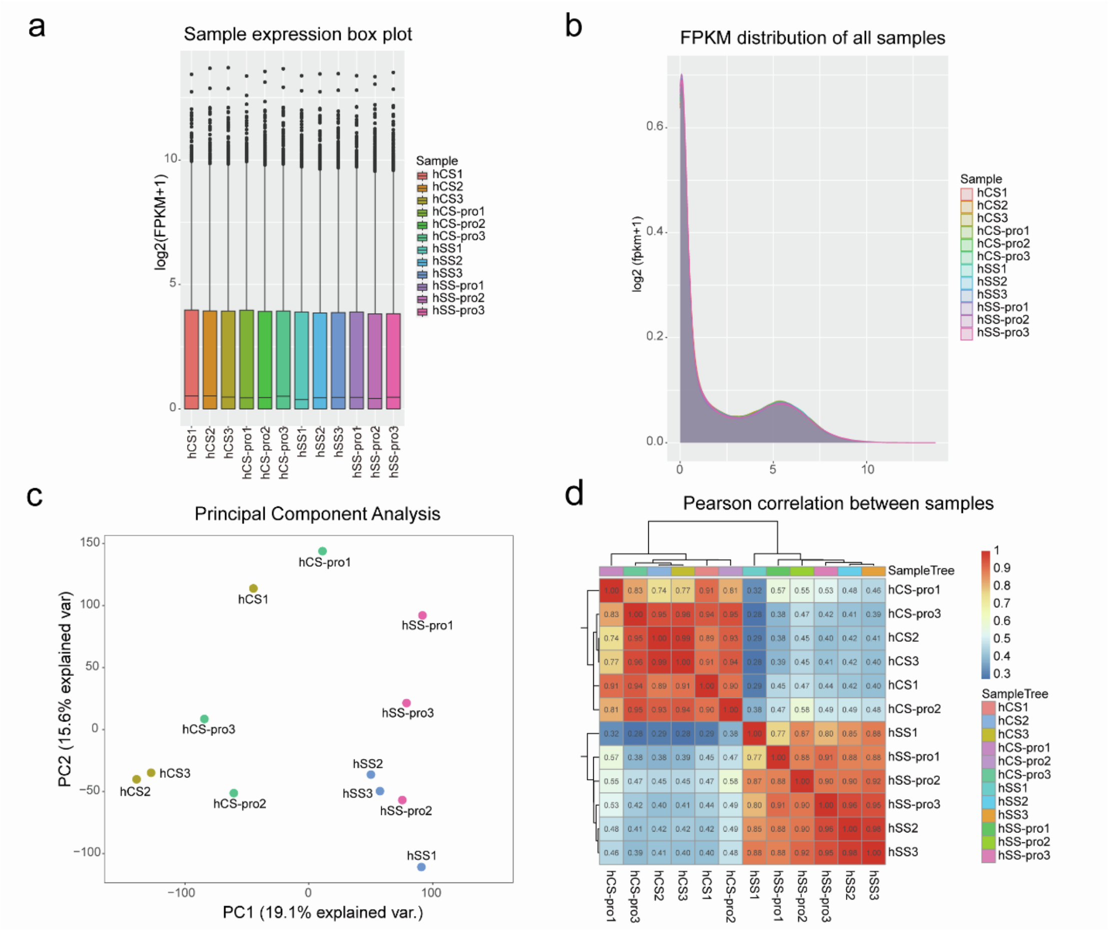

Figure S1. Gene Expression Distribution, Principal Component Analysis, and Sample Correlation Overview. (a) Box plot demonstrating the mRNA gene expression abundance in each sample. (b) Fragments Per Kilobase of transcript per Million mapped reads (FPKM) (mRNA expression abundance) distribution of all samples. (c) Principal component analysis (PCA) of the samples (based on mRNA gene expression). (d) Heatmap showing the correlation analysis of mRNA gene expression abundance in each sample.

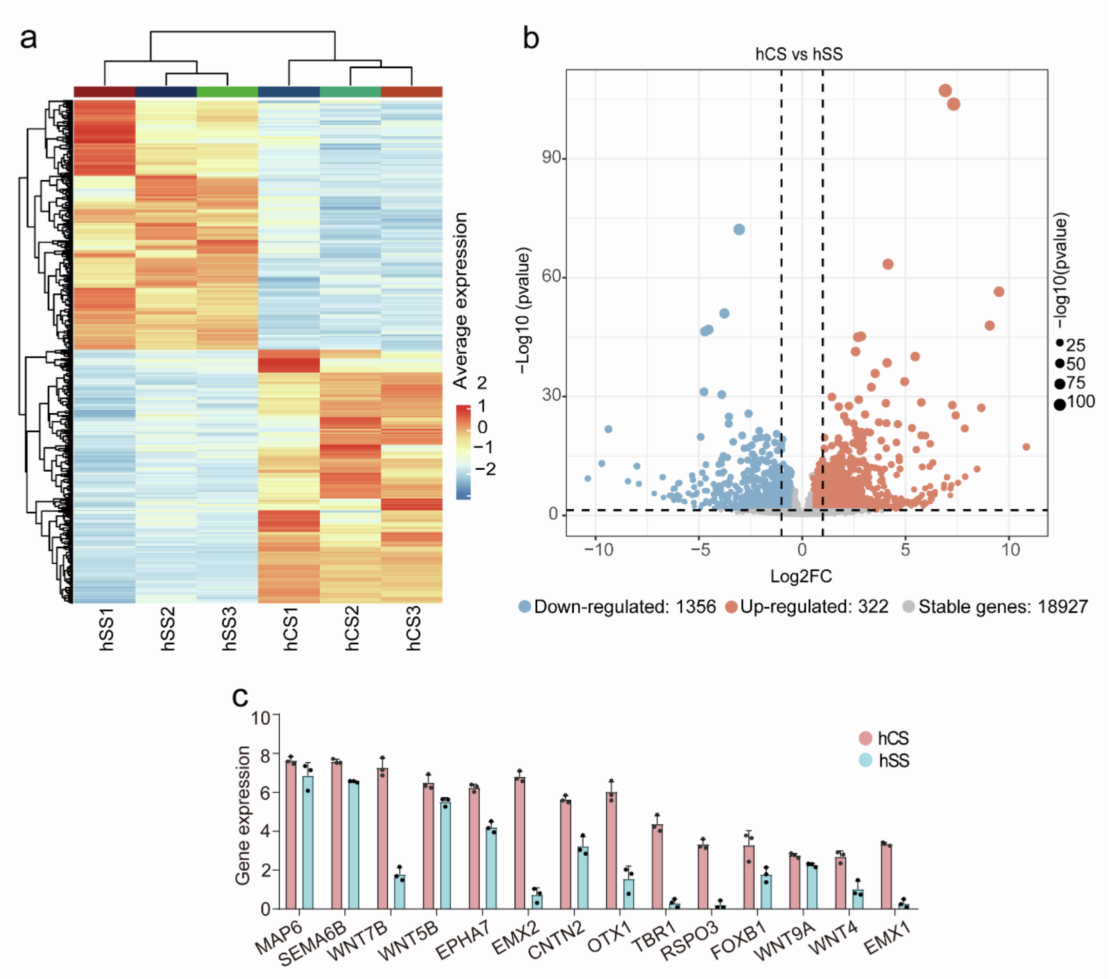

Figure S2. Differentially expressed genes between the hCS and hSS groups. (a) Heatmap showing the average expression levels of DEGs in hCS and hSS samples (red indicates high expression, blue indicates low expression). (b) Volcano plot illustrating DEGs between hCS and hSS, with upregulated genes (red), downregulated genes (blue), and stable genes (gray). (c) Bar plots displaying expression levels of dorsal forebrain markers (EMX1, EMX2, OTX1) and Wnt pathway activators (RSPO3, WNT7B, WNT5B) in hCS and hSS.

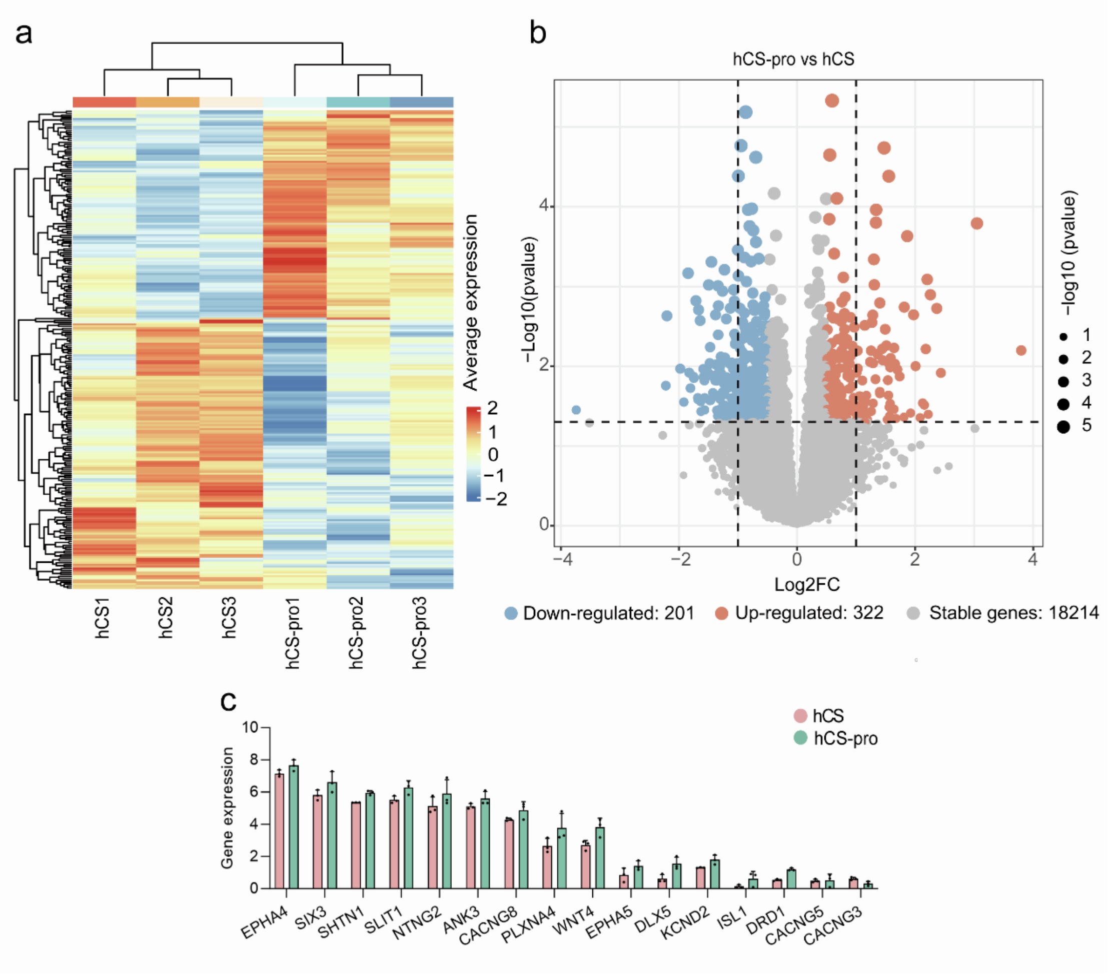

Figure S3. Differentially expressed genes between the hCS-pro and hCS groups. (a) Heatmap showing average expression levels of DEGs in hCS-pro and hCS samples (red indicates high expression, blue indicates low expression). (b) Volcano plot illustrating DEGs between hCS-pro and hCS, with upregulated genes (red), downregulated genes (blue), and stable genes (gray). (c) Bar plots displaying expression levels of key genes like DRD1, KCND2, and SLIT1 in hCS-pro and hCS.

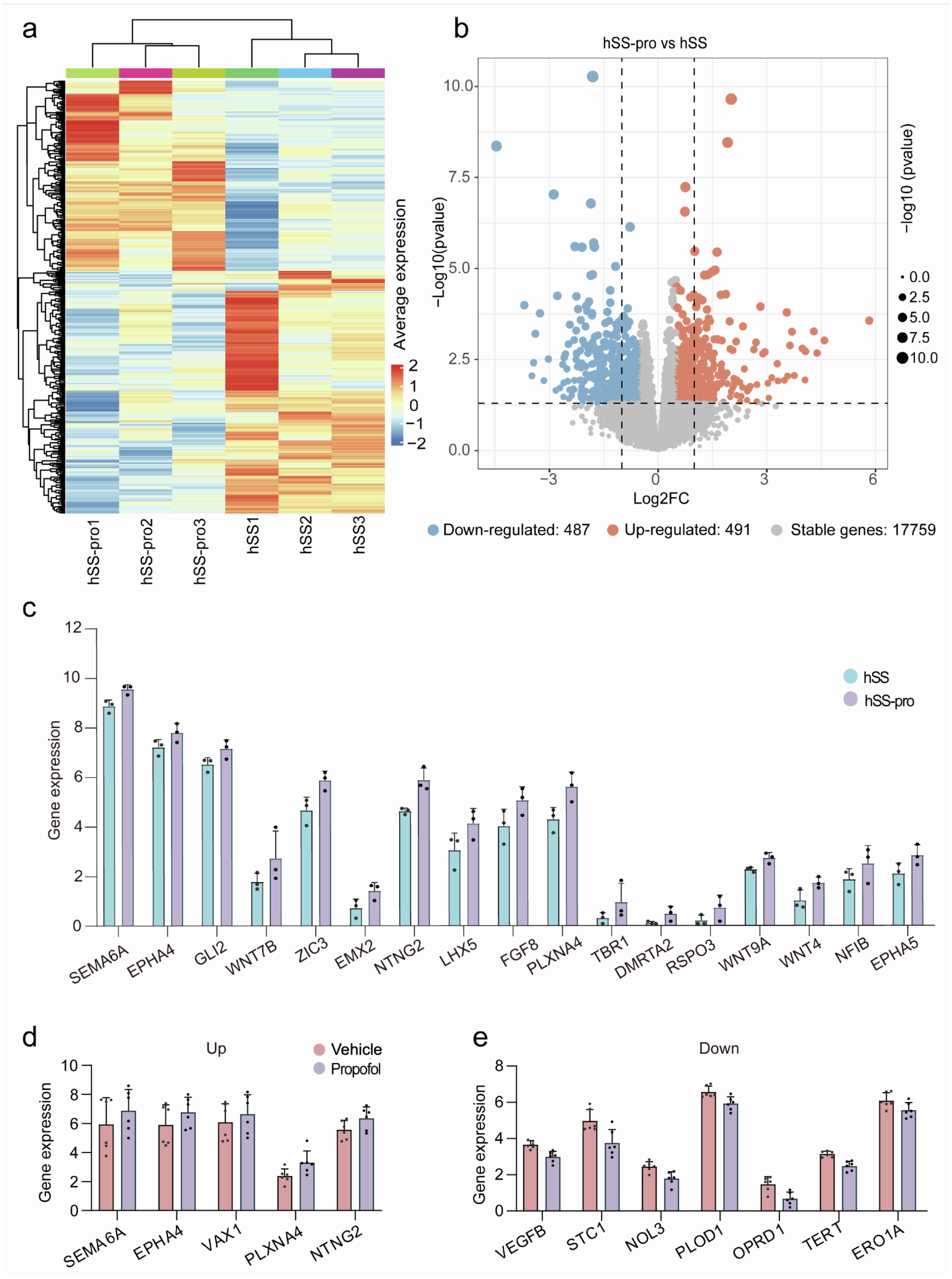

Figure S4. Differentially expressed genes between the hSS-pro and hSS groups, and between the vehicle and propofol groups.

(a) Heatmap showing average expression levels of DEGs in hSS-pro and hSS samples (red indicates high expression, blue indicates low expression). (b) Volcano plot illustrating

DEGs between hSS-pro and hSS, with upregulated genes (red), downregulated genes (blue), and stable genes (gray). (c) Bar plots displaying expression levels of key genes like FGF8, EMX2, and PAX6 in hSS-pro and hSS. (d) Expression levels of key upregulated genes, such as PLXNA4 and WNT4, involved in axon guidance. (e) Expression levels of downregulated genes, including VEGFB and PFKFB3, associated with hypoxia and metabolism.

Table

Antibody	Host	Dilution	Source	Catalog/Clone No.
Cleaved Caspase-3	Rabbit	1:400	Cell Signaling Technology	9661S
TUBB3	Mouse	1:1000	BioLegend	801201
SOX2	Mouse	1:500	R&D Systems	MAB2018
FOXG1	Rabbit	1:1000	Abcam	ab196868
NEUN	Mouse	1:200	Millipore	MAB377
MAP2	Chicken	1:500	Abcam	ab5392
NKX2.1	Rabbit	1:500	Abcam	ab76013

Supplemental Table 1

Primer	Sequence (5'→3')
SOX2-F	5'-TGGACAGTTACGCGCACAT-3'
SOX2-R	5'-CGAGTAGGACATGCTGTAGGT-3'
PAX6-F	5'-TGGGCAGGTATTACGAGACTG-3'
PAX6-R	5'-ACTCCCGCTTATACTGGGCTA-3'
MAP2-F	5'-CGAAGCGCCAATGGATTCC-3'
MAP2-R	5'-TGAACTATCCTTGCAGACACCT-3'
VGLUT1-F	5'-CAGAGTTTTCGGCTTTGCTATTG-3'
VGLUT1-R	5'-GCGACTCCGTTCTAAGGGTG-3'
GABBR2-F	5'-ACCAACTTCTTCGGGGTCAC-3'
GABBR2-R	5'-CACCTCCCTGCTGTCTTGAA-3'
VIM-F	5'-GACGCCATCAACACCGAGTT-3'
VIM-R	5'-CTTTGTCGTTGGTTAGCTGGT-3'
Caspase-3-F	5'-CCAAAGATCATACATGGAAGCG-3'
Caspase-3-R	5'-CTGAATGTTTCCCTGAGGTTTG-3'
GAPDH-F	5'-CATGAGAAGTATGACAACAGCCT-3'
GAPDH-R	5'-AGTCCTTCCACGATACCAAAGT-3'

Supplemental Table 2

Methods

Generation from hiPSC of hCS and hSS

Remove the maintenance medium from the hiPSC culture six-well plate 1 hour prior to use. Add 2 mL of Stem Flex medium (Gibco, A33493-01) supplemented with Y-27632 (10 μM; Selleck Chemicals, S1049) to each plate. Prepare the AggreWell 24-well plate by adding 1 mL of medium, supplemented with Y-27632, to each well. Centrifuge the AggreWell plate at 2,000g for 5 minutes using a swinging bucket rotor with a plate holder to remove air bubbles from the microwells.

Remove the maintenance medium from the hiPSC culture plates and wash the cells once with PBS (MesGen, MG3150-500ml). Add 1 mL of Accutase (STEMCELL Technologies, 07920) to each plate and incubate at 37°C for 6 minutes in a 5% CO_2 incubator, gently shaking the plates until the cells detach. Add 1 mL of DMEM/F12 (Gibco, 11330032) to terminate digestion and transfer the cell suspension to a centrifuge tube containing 5 mL of DMEM/F12. Centrifuge the suspension at 300g for 3 minutes. Resuspend the cell pellet in medium supplemented with Y-27632 to achieve a final concentration of 3 million cells per mL. Transfer 1 mL of this cell suspension into each well of the AggreWell plate to achieve a final volume of 2 mL per well. Centrifuge the AggreWell plate at 100g for 3 minutes. Incubate the cells at 37°C, 5% CO_2 for 26 hours.

Collect spheroids from the microwells by gently pipetting the medium up and down two to three times using a micropipette with a 1,000-μL tip. Transfer the collected spheroids to ultra-low-attachment 10-cm culture dishes. Supplement the medium with dorsomorphin (5 μM; Selleck Chemicals, s7840) and SB-431542 (10 μM; Selleck Chemicals, s1067). For

the first five days, the hiPSC medium was changed daily and supplemented with dorsomorphin and SB-431542. On the D6, neural spheroids in suspension were transferred to neural medium (NM) containing Neurobasal (Gibco, 21103049), B-27 supplement without vitamin A (Gibco, 12587001), GlutaMax (1:100; Gibco, 35050061), penicillin and streptomycin (1:100; Life Technologies, 15140122), and supplemented with growth factors EGF (20 ng/mL; NovoProtein, C029) and FGF2 (20 ng/mL; NovoProtein, C046) until D24. For the generation of hSS, the medium was supplemented with additional small molecules during the first D23 of culture. The hSS condition included the addition of the Wnt pathway inhibitor IWP-2 (5 μM; Selleckchem, S7085) from D4 to D23, and the SHH pathway agonist SAG (smoothened agonist; 100 nM; Selleckchem, S7779) from D12 to D23. AlloP (Allopregnanolone; 100 nM, Selleck, S5805) was added from D15 to D23, and there was a brief exposure (D12–15) to retinoic acid (RA, 100 nM; Sigma, R2625). The medium was changed every other day from D15 onwards. From D25 to D42, the NM for both the hCS and hSS conditions was supplemented with growth factors BDNF (20 ng/ml; NovoProtein, C076) and NT3 (20 ng/ml; NovoProtein, C079). From D43 onwards, hCS and hSS were maintained in unsupplemented NM with medium changes every four days. The hCS were cultured in the same manner as hSS, except for the addition of WP-2, SAG, AlloP, and RA.

Experimental Groups

Both hCS and hSS organoids were exposed to 20 μM propofol for 6 hours on day 11 (D11). The propofol-treated groups were compared to control groups, which were treated

with vehicle (solvent) only.

Experimental Timeline: Organoids were harvested at different time points to assess changes at different developmental stages. Morphological analyses were performed at D11, D18, and D25, while immunofluorescence and PCR were carried out at D18, D25, D40, and D80. Electrophysiological analyses were conducted between D40 and D70, and RNA-seq was performed at D18 to evaluate transcriptional changes.

Experimental Replication: The experiments were performed in biological replicates, with each condition having at least three independent organoid cultures to ensure reproducibility and reliability of the results.

Bulk RNA sequencing (RNA-Seq) and bioinformatics analysis

Four groups of brain organoids derived from hiPSCs, with a minimum of 15 organoids in each group, were analyzed. Total RNA from the brain organoids was extracted using the TRIzol reagent (Invitrogen, Thermo Fisher Scientific) and subjected to library construction and high-throughput RNA sequencing on the Illumina sequencing platform. DEGs between the groups were identified using DESeq2 (version 1.20.0). A threshold of an adjusted p-value < 0.05 and an absolute log2 fold change ≥ 0.5 was applied. Gene ontology (GO) enrichment analysis of the DEGs and statistical enrichment analysis of the Kyoto Encyclopedia of Genes and Genomes (KEGG) pathways were performed using the clusterProfiler software (version 3.8.1).

Viral labeling of neural spheroids

Briefly, hCS were transferred into a 1.5 mL microcentrifuge tube containing 400 μL of NM supplemented with 0.4 μL of recombinant adeno-associated virus (rAAV-hSyn-EGFP-WPRE-hGH pA), which expresses enhanced green fluorescent protein (EGFP) under the control of the human synapsin promoter. The spheroids were incubated overnight at 37°C with 5% CO_2 to promote viral infection. The following day, the infected neural spheroids were transferred to fresh NM.

Cryopreservation

hCS or hSS were fixed in 4% paraformaldehyde (Solarbio, P1110) for 1 hour, followed by washing with PBS. They were then transferred to a 30% sucrose solution and incubated at 4°C overnight for 48 hours. Subsequently, the spheroids were embedded in Tissue-Tek OCT compound (Sakura Finetek, 4583), snap-frozen on dry ice, and stored at -80°C. For immunohistochemistry, 20 μm thick sections were prepared using a cryostat (Leica).

Immunohistochemistry

Cryosections were washed with PBS to remove excess OCT and blocked in 5% normal goat serum (NGS, sangon biotech, E510009) and 0.3% Triton X-100 (sigma, T8787) in PBS for 1 hour at room temperature. The sections were then incubated overnight (16 hours) at 4°C with primary antibodies diluted in PBS containing 2% NGS and 0.3% Triton X-100. After washing off the primary antibodies with PBS, the cryosections were incubated with secondary antibodies diluted in PBS with 2% NGS and 0.3% Triton X-100 for 1 hour.

Following another PBS wash, the sections were stained with DAPI (1:1000 in PBS, Selleck Chemicals, S2215) for 10 minutes to visualize nuclei. The primary antibodies used were anti-Cleaved Caspase-3 (rabbit, 1:400; Cell Signaling Technology: 9661S), anti-TUBB3 (mouse, 1:1000; BioLegend: 801201), and anti-SOX2 (mouse, 1:500; R&D Systems: MAB2018). The sections were mounted on glass slides using Aquamount (Thermo Scientific) and imaged using an Olympus SpinSR10 confocal microscope. The antibodies used are listed in Supplementall Table 1.

Real time quantitative PCR (qPCR)

mRNA was isolated using Trizol, and template cDNA was synthesized by reverse transcription using the SuperScript III First-Strand Synthesis SuperMix for qRT-PCR (Thermo Fisher, 11752050). GAPDH was applied as housekeeping gene. Real-time qPCR was performed using SYBR Green (Roche) on a ViiA7 machine (Applied Biosystems, Life Technologies). Data were processed using the QuantStudio RT-PCR software (Applied Biosystems). Primers used are listed in Supplemental Table 2.

Electrophysiology

Sections of hCS (D40–70) for physiological recordings were obtained using a previously described approach. Briefly, hCS were embedded in 0.4% low-melting agarose (Takara, 5264), diluted with ACSF, and sectioned using a Leica VT1200S vibratome. Spheroids were incubated in bicarbonate-buffered artificial cerebrospinal fluid (aCSF) at 32°C and equilibrated with a mixture of 95% O_2 and 5% CO_2. The aCSF solution contained

126 mM NaCl, 26 mM $NaHCO_3$, 10 mM glucose, 2.5 mM KCl, 1.25 mM NaH_2PO_4, 1 mM $MgSO_4$, and 2 mM $CaCl_2$. Slicing was performed using a Leica VT1200S vibratome. Immediately after sectioning, slices were transferred to a circulation chamber containing oxygenated aCSF at 32°C. For patch-clamp recording, cells were identified by the presence of a fluorescent reporter using an upright BX51W1 microscope (Olympus). Recording electrodes made of borosilicate glass had a resistance of 4-9 MΩ when filled with internal solution. The low-Cl internal solution contained 145 mM K+ gluconate, 0.1 mM $CaCl_2$, 2.5 mM $MgCl_2$, 10 mM HEPES, 0.2 mM EGTA, and 4 mM Na+ phosphocreatine. Data were collected using a 1550B digitizer (Molecular Devices), and a 700B patch-clamp amplifier (Molecular Devices), and acquired with Clampex 10.7 software (Molecular Devices). Recordings were filtered at 10 kHz. APs were analyzed using Clampfit 10.7.0.3 programs.
